# The prevalence of vitamin A deficiency and its public health significance in children in low- and middle-income countries: A systematic review and modelling analysis

**DOI:** 10.7189/jogh.13.04084

**Published:** 2023-08-11

**Authors:** Peige Song, Davies Adeloye, Shuting Li, Dong Zhao, Xinxin Ye, Qing Pan, Yiwen Qiu, Ronghua Zhang, Igor Rudan, Global Health Epidemiology Research Group (GHERG)

**Affiliations:** 1 School of Public Health and Women's Hospital, Zhejiang University School of Medicine, Hangzhou, Zhejiang, China; 2 Centre for Global Health Research, Usher Institute of Population Health Sciences and Informatics, University of Edinburgh, Edinburgh, Scotland, UK; 3 School of Public Health, Zhejiang University School of Medicine, Hangzhou, Zhejiang, China; 4 Zhejiang Provincial Centre for Disease Control and Prevention, Hangzhou, Zhejiang, China; 5 College of Education, Zhejiang University, Hangzhou, Zhejiang, China; 6 Algebra University, Zagreb, Croatia

## Abstract

**Background:**

Vitamin A deficiency (VAD) is widely recognised as a major public health concern in low- and middle-income countries (LMICs). Despite various interventions implemented in many countries, a lack of reliable data is hindering progress. We aimed to consolidate available data and quantify estimates of the prevalence of VAD among children ≤18 years in LMICs.

**Methods:**

We searched PubMed, Medline and Embase for studies reported the prevalence of VAD or marginal (m)VAD among children. A multilevel mixed-effects meta-regression approach was applied to establish the regression models for VAD and mVAD prevalence. The total numbers of children affected by VAD and mVAD in LMICs in 2019 were separately calculated from the estimated age- and socio-demographic index (SDI)-specific prevalence with their corresponding United Nations Population Division populations projections. We estimated areas of significant public health concern in 165 LMICs using the lower confidence interval (CI) of VAD prevalence.

**Results:**

A total of 116 articles from 40 LMICs were retained. In 2019, VAD and mVAD affected 333.95 million (95% CI = 253.00-433.74) and 556.13 million (95% CI = 388.83-767.94) children and adolescents in 165 LMICs, respectively, corresponding to a prevalence of 14.73% (95% CI = 11.16-19.14) and 24.54% (95% CI = 17.15-33.88). The prevalence of both VAD and mVAD was the highest in children aged 0-5 years at 19.53% (95% CI = 15.03-24.91) and 28.22% (95% CI = 20.00-38.24), respectively, with both steadily decreasing to 10.09% (95% CI = 7.44-13.50) and 20.76% (95% CI = 14.16-29.50) in adolescents aged 13-18 years. The prevalence of VAD was significantly higher in the low SDI region at 29.67% (95% CI = 22.67-37.53) compared to 5.17% (95% CI = 3.14-8.43) estimated in the high-middle SDI region. 68 of the 165 LMICs (41.21%) were classified as areas of moderate to severe VAD public health significance.

**Conclusions:**

VAD continues to pose a significant public health concern in many low-income settings. Development in LMICs is a crucial factor for VAD, with a disproportionately higher burden in low SDI regions.

**Registration:**

This study protocol was registered with PROSPERO, CRD42020220654.

Vitamin A deficiency (VAD) is widely recognised as a major public health concern across many low- and middle-income countries (LMICs) [1]. It is widely acknowledged as a leading micronutrient deficiency with adverse impacts on pregnancy, child growth and development [2,3]. Approximately one-third of children under the age of five have VAD, contributing to approximately 2% of deaths in this age group [1,4].

Vitamin A, a fat-soluble vitamin derived from animal or plant sources in the diet, is a crucial micronutrient that plays essential roles in body physiologic functions, including vision, cognition and alertness, cell division, reproduction, and bone development, while also reducing risk of common childhood infections like diarrhoea, measles and respiratory diseases [3]. Of note, VAD can also result in xerophthalmia, a clinical manifestation of VAD that includes night blindness, corneal scarring, and Bitot’s spots. In extreme cases, severe VAD can cause permanent blindness [5]. The World Health Organization (WHO) estimates that up to 500 million children are blind due to VAD, with half dying within a year of losing their vision [4]. Serum retinol levels are often used to assess the status of Vitamin A in the body, with concentrations below 0.70 micromole per litre (μmol / l) suggestive of VAD [4]. The primary cause of VAD is insufficient dietary intake to satisfy physiological needs, although serum levels could also be affected by high rate of infections [4].

Although children are the most affected population, VAD commonly occurs in clusters among families and communities, indicating that many individuals in such clusters, including older children and adults, may have subclinical or marginal deficiencies [5]. To address this deficiency in vulnerable settings especially those with high prevalence of night blindness, the WHO recommends periodic vitamin A supplementation (VAS) for children aged 6-59 months [4]. Despite widespread implementation, including supplementation, fortification and diversified diets, in approximately 100 countries globally, hundreds of millions of preschool children are still affected, mostly residing in sub-Saharan Africa and South-East Asia Region (SEAR) [1].

The high levels of food insecurity, poverty, and illiteracy in many LMICs have continued to drive poor child feeding practices, leading to widespread essential micronutrient deficiencies like vitamin A [6]. Despite notable improvements in these driving factors over the past decade in high-income countries (HICs) and some middle-income countries, low-income countries remain grossly affected. The prevalence data for VAD at the national levels in many LMICs is limited, incomplete, or missing, and many countries have national VAS programme supported by little or no VAD prevalence data [2]. The burden of VAD has decreased in many HICs, but remains high in Central Africa and South Asia, where knowledge and awareness are limited [7].

The 2009 WHO report [4] and 2013 global study by Stevens et al. [1] provide important information on the epidemiology of VAD, and the Institute for Health Metrics and Evaluation have continued to provide annual updates as part of the global burden of disease (GBD) project [7,8]. Despite these efforts, there is a need for continued understanding of the epidemiology of VAD in many LMICs in order to support many ongoing local and international efforts targeted at child malnutrition and VAD [9]. In this study, we have comprehensively reviewed publicly available evidence on the prevalence of VAD in LMICs to provide updated estimates of the prevalence in regions and countries across these settings, and to highlight areas where the burden is of significant public health concern to raise awareness and guide targeted response.

## METHODS

The study conforms to the Preferred Reporting Items for Systematic reviews and Meta-Analysis (PRISMA) [10] and Meta-analysis of Observational Studies in Epidemiology guidelines [11]. This study protocol was registered with PROSPERO, CRD42020220654.

### Search strategy and selection criteria

A comprehensive literature search was conducted in PubMed, MEDLINE and Embase to identify English language articles published between January 1, 2000, and October 5, 2022 that reported the prevalence of VAD or marginal (m)VAD, either alone or in combination, in children aged 18 years or younger worldwide. No language or geographic restrictions were applied. Search terms used were combinations of VAD or symptoms (e.g., “vitamin A deficiency”, “night blindness”, “xerophthalmia”), children (e.g., “children”, “adolescents”, “paediatric”), and prevalence (e.g., “prevalence”, “epidemiology”). The detailed search strategies for each database are listed in Table S1 in the **Online Supplementary Document**. Reference lists of the included studies were reviewed to identify additional eligible studies. Titles and abstracts retrieved by searches were screened independently by two authors (QP and YQ). Full-text articles were also reviewed independently by the same two authors. Any disagreements were resolved through a joint meeting with a senior author (XY) to reach a consensus.

Eligible studies were observational population-based studies that investigated the prevalence of VAD / mVAD in children and adolescents aged 18 years and younger in the general population. Investigations could have been organised in communities, schools or health care facilities (only in forms of regular health check-ups). To largely reduce the heterogeneity caused by case definitions, vitamin A status should have been assessed based on serum (or plasma) retinol concentration as recommended by WHO [4], with a cut-off of <0.70 μmol / l (<20 micrograms (μg) / decilitres (dl)) to define VAD, and of between 0.70 μmol / l and 1.05 μmol / l (20 μg / dl-30 μg / dl) to define mVAD [12]. Studies were excluded if the investigated samples were unrepresentative of the general paediatric population, such as wasting or stunting children. Moreover, studies were not eligible if they defined VAD or mVAD based on retinol-binding protein concentration or self-reports. Studies that did not report the uncertainty of prevalence estimates, such as confidence interval (CI) or standard error (SE), or did not have enough information on sample size and number of cases for computing uncertainty were also excluded. In case where a study’s findings were reported by more than one article, we gave preference to the article with the most comprehensive results.

### Data extraction and quality assessment

Using a piloted data extraction form, two authors (QP and YQ) extracted and cross-checked data on the following variables: 1) study characteristics: first author, year of publication, study setting (e.g., community-based, school-based), study location, country of data collection, geographic region as designated by WHO (i.e., African Region (AFR), Region of the Americas (AMR), SEAR, European Region (EUR), Eastern Mediterranean Region (EMR), and Western Pacific Region (WPR)), development region according to the socio-demographic index ((SDI), a summary measure of overall development constructed from income per capita, average years of schooling, and total fertility rate) in 2019 as HICs (high SDI = SDI ≥0.805) group and LMICs (high-middle SDI = 0.690-0.804; low-middle SDI = 0.455-0.689; low SDI = <0.455) [13], study period, study design, test method of retinol concentration; 2) population characteristics: sample size, age (mean, median, or range), the proportion of girls, number of VAD / mVAD cases. Where available, prevalence data were extracted by age group and sex from the same study. Where not available, prevalence for the overall children group and the corresponding proportion of girls were extracted. In case of censoring age groups, such as people aged 12 years and above, we imputed the missing band by taking the same (or average) width reported in the same article. Otherwise, when no other age groups were reported, we imputed it as 18 years when the study population was explicitly confined to children. Based on the mean time-lag between investigation and publication from articles with available information showed in Table S2 in the **Online Supplementary Document**, we imputed the years of investigation as five years before the publication years for articles without such information.

In parallel, two authors (XY and YQ) independently assessed the quality of included articles using the Quality Assessment Tool with five modules, namely sample population, sample size, participation rate, outcome assessment, and analytical methods [14]. Each module was rated as zero (low quality), one (moderate quality) or two (high quality). The overall scores range between zero and 10. The detailed quality assessment scale are provided in Table S3 in the **Online Supplementary Document**.

### Statistical analysis

The focus of this study was on LMICs, with prevalence estimations made for VAD and mVAD in 165 LMICs.

#### Epidemiological modelling of the prevalence of VAD and mVAD in children

Due to the hierarchical structure of extracted data, we applied a multilevel mixed-effects meta-regression approach to establish the regression models of VAD and mVAD prevalence. The effect of data points clustering from the same study was controlled by adding the study identification and country identification into the regression model as the random effect () [15].

Given that:

prevalence of VAD / mVAD = p = *(VAD / mVAD cases) ⁄ (number of participants)*

Then, the prevalence was stabilised with the logit link,

logit(p) = ln(*p* ⁄ [1 – *p*]) = ln(*odds*) = *α* + *β*_1_ * *x*_1_ + *β*_2_ * *x*_2_ + ⋯ + *u_i_*

*x*_1_ – *x*_n_ were cluster-level variables, including age, sex (boys vs. girls) or the proportion of girls, setting (rural vs. urban), investigation site (community-based or facility-based (school-based and health check-ups)), SDI (categorical as high-middle SDI vs. low-middle and low SDI), WHO region, investigation period (categorical as before 2000, 2000-2009, 2010 and later), retinol test method. The associations of cluster-level variables were first examined using an age-adjusted meta-regression, the results are shown in Table S4 in the **Online Supplementary Document**. The prevalence of VAD was found to be negatively associated with age and SDI, decreased in recent decades, and higher among boys (than among girls), in rural areas (than in urban areas), in low-middle and low SDI region (than in high-middle SDI region), in AFR (than in WPR). While the prevalence of mVAD significantly decreased with increased age, and was higher in rural areas (than in urban areas). In the multivariable models, age and SDI were included in the regression models of VAD prevalence. Therefore,

logit(p) = *α + β1* x *Age + β2* x SD*I + ui*

Then,

prevalence of VAD / mVAD = *p* = e^(α + β₁^ ^x Age + β₂^ ^x SDI + uᵢ)^ ⁄ (1 + e^(α + β₁^ ^x Age + β₂^ ^x SDI + uᵢ)^)

#### Estimation of the national prevalence of VAD and mVAD in children in 2019

Based on the above models, the age- and SDI-specific prevalence estimates of VAD and mVAD were respectively estimated. First, the total numbers of children affected by VAD and mVAD (“VAD envelope” and “mVAD envelope”) in LMICs were respectively calculated by multiplying the estimated age- and SDI-specific prevalence rates by the corresponding paediatric populations in 2019, obtained from the United Nations Population Division [16]. Then to take into account the effect of urbanisation, the odds ratios of urban areas (vs. rural areas) for VAD and mVAD as generated in step one were obtained, and a risk-factor-based model was used to distribute the total numbers of children affected by VAD and mVAD in LMICs into the 165 LMIC nations [17,18]. Finally, the national prevalence of VAD and mVAD was generated by dividing the number of VAD and mVAD cases by the corresponding paediatric population in 2019.

#### Classification of VAD as a problem of public health significance in 2019

The public health significance of VAD in 165 LMICs was evaluated based on the lower CI of VAD prevalence given the uncertainties in point prevalence estimates. With reference to the WHO guide on VAD public health significance [4], we classified a lower CI of VAD prevalence of ≥2% but <10% as mild; ≥10% but <20% as moderate; and ≥20% as severe areas of public health significance, respectively.

More details on the statistical analysis are provided in the eMethods in the **Online Supplementary Document**. All analyses were performed using R, version 3.3.0 (R Foundation for Statistical Computing, Vienna, Austria). The significance level was set as two-sided *P* < 0.05 for all analyses.

## RESULTS

**Figure 1:**
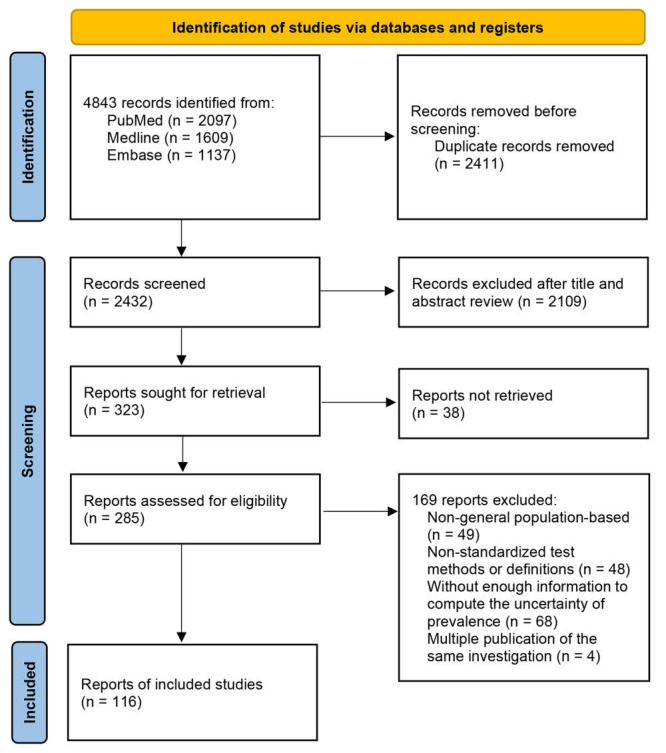
Flowchart of study selection. VAD – vitamin A deficiency, mVAD – marginal vitamin A deficiency

A total of 4843 records were identified through searches of bibliographic databases. After removing 2411 duplicates and 2109 irrelevant records after title and abstract review, 116 eligible articles were retained. This covered 286 128 children and adolescents from a total of 40 LMICs (**Figure 1**). Of these articles, 114 articles reported data on the prevalence of VAD, and 44 reported the prevalence of mVAD. A total of 74 articles were community-based studies (64%), 24 (21%) were school-based, and 18 (16%) were based on health check-ups. The detailed characteristics and quality scores of the included articles are shown in the Table S5 in the **Online Supplementary Document**. The geographic locations of the 116 included articles are shown in Figure S1 in the **Online Supplementary Document**.

The associations between age and prevalence of VAD and mVAD were constructed based two sets of data points (257 for VAD and 86 for mVAD; Table S4 and Figure S2 in the **Online Supplementary Document**). Generally, the prevalence of VAD and mVAD was lower with advanced age and higher SDI. This decreasing trend with age was more pronounced for VAD than for mVAD. After adjusting for the demographics and urbanisation progresses in 165 LMICs in 2019, the overall and regional prevalence and cases of VAD and mVAD among children was estimated.

In 2019, VAD affected 333.95 million (95% CI = 253.00-433.74) children and adolescents, which corresponded to 14.73% (95% CI = 11.16-19.14) of the paediatric population in 165 LMICs (**Table 1**). Across different age groups, the prevalence of VAD was the highest in children aged 0-5 years (19.53%, 95% CI = 15.03-24.91), then became steadily lower reducing to 10.09% (95% CI = 7.44-13.50) in adolescents aged 13-18 years. Across different development regions, the prevalence of VAD gradually decreased with SDI. Regional estimates based on development levels indicated that the highest prevalence of VAD was 29.67% (95% CI = 22.67-37.53) in low SDI region, a value that was almost five times higher than estimated in high-middle SDI region (5.17%, 95% CI = 3.14-8.43). The largest child cases of VAD were in low SDI region-a total of 138.59 million (95% CI = 105.89-175.30). Notably, more than one-third of children aged 0-5 years were affected by VAD in low SDI region (36.87%, 95% CI = 28.82-45.46), translating to 62.29 million (95% CI = 48.69-76.80) cases. By geography, AFR had the largest share of VAD cases (135.03 million, 95% CI = 103.78-170.89) and the highest prevalence of VAD in children (24.51%, 95% CI = 18.84-31.02), while EUR had the least VAD cases (8.49 million, 95% CI = 5.43-13.28) as well as the lowest prevalence (5.89%, 95% CI = 3.76-9.22). Children aged 0-5 years in AFR were the group with highest prevalence of VAD (30.59%, 95% CI = 23.99-37.83) and most VAD cases (61.92 million, 95% CI = 48.56-76.59).

**Table 1:** The estimated prevalence and number of cases of vitamin A deficiency (VAD) among children residing in low- and middle-income countries (LMICs) in 2019, by age group

Region	0-5 years		6-12 years		13-18 years		0-18 years	
	**Prevalence (%)**	**Cases (million)**	**Prevalence (%)**	**Cases (million)**	**Prevalence (%)**	**Cases (million)**	**Prevalence (%)**	**Cases (million)**
**LMIC overall**	19.53 (15.03-24.91)	146.68 (112.9-187.06)	14.18 (10.70-18.51)	119.10 (89.83-155.44)	10.09 (7.44-13.50)	68.17 (50.27-91.25)	14.73 (11.16-19.14)	333.95 (253.00-433.74)
**Development region**								
High-middle SDI	7.00 (4.27-11.27)	4.00 (2.44-6.44)	4.95 (3.00-8.10)	3.32 (2.01-5.43)	3.47 (2.08-5.75)	1.83 (1.10-3.04)	5.17 (3.14-8.43)	9.15 (5.55-14.91)
Middle SDI	10.99 (7.76-15.37)	28.29 (19.96-39.55)	7.85 (5.48-11.15)	22.95 (16.00-32.59)	5.50 (3.78-7.95)	13.18 (9.06-19.04)	8.16 (5.71-11.55)	64.43 (45.03-91.18)
Low-middle SDI	19.50 (15.65-24.05)	52.18 (41.88-64.36)	14.17 (11.21-17.77)	43.80 (34.65-54.94)	10.12 (7.85-12.96)	25.99 (20.16-33.28)	14.63 (11.60-18.30)	121.97 (96.69-152.57)
Low SDI	36.87 (28.82-45.46)	62.29 (48.69-76.80)	28.63 (21.70-36.48)	49.09 (37.22-62.56)	21.49 (15.78-28.38)	27.21 (19.98-35.94)	29.67 (22.67-37.53)	138.59 (105.89-175.30)
**WHO region**								
AFR	30.59 (23.99-37.83)	61.92 (48.56-76.59)	23.43 (17.88-29.86)	47.70 (36.40-60.80)	17.55 (13.00-23.14)	25.42 (18.82-33.51)	24.51 (18.84-31.02)	135.03 (103.78-170.89)
AMR	12.28 (9.10-16.42)	7.65 (5.67-10.22)	8.82 (6.45-11.98)	6.43 (4.71-8.73)	6.23 (4.48-8.61)	3.99 (2.87-5.51)	9.07 (6.65-12.28)	18.07 (13.24-24.47)
EMR	22.07 (17.15-27.72)	22.40 (17.41-28.13)	16.73 (12.70-21.50)	17.65 (13.41-22.69)	12.45 (9.22-16.44)	9.90 (7.32-13.07)	17.43 (13.31-22.3)	49.94 (38.14-63.89)
EUR	8.04 (5.20-12.38)	3.81 (2.46-5.86)	5.60 (3.56-8.81)	3.07 (1.95-4.83)	3.85 (2.41-6.19)	1.62 (1.01-2.59)	5.89 (3.76-9.22)	8.49 (5.43-13.28)
SEAR	17.62 (13.85-22.19)	35.89 (28.21-45.22)	12.83 (9.96-16.41)	31.78 (24.67-40.65)	9.22 (7.04-12.02)	20.10 (15.34-26.20)	13.11 (10.19-16.74)	87.77 (68.22-112.07)
WPR	11.29 (7.97-15.79)	15.10 (10.67-21.12)	8.07 (5.63-11.47)	12.53 (8.74-17.81)	5.65 (3.88-8.18)	7.19 (4.94-10.42)	8.36 (5.85-11.85)	34.82 (24.35-49.34)

VAD – vitamin A deficiency, LMICs – low- and middle-income countries, SDI – socio-demographic index, WHO – The World Health Organization, AFR – African Region, AMR – Region of the Americas, SEAR – South-East Asia Region, EUR – European Region, EMR – Eastern Mediterranean Region, WPR – Western Pacific Region

As listed in **Table 2**, the prevalence of mVAD was 24.54% (95% CI = 17.15-33.88) in LMICs, which corresponded to a total of 556.13 million (95% CI = 388.83-767.94) affected children and adolescents. The prevalence of mVAD was the highest in children aged five years and younger (28.22%, 95% CI = 20.00-38.24), then slowly decreased in older children, and reached to 20.76% (95% CI = 14.16-29.50) in adolescents aged 13-18 years. Regionally, the prevalence of mVAD was the highest in low SDI region (33.40%, 95% CI = 20.70-49.48), while the most cases of mVAD was observed in low-middle SDI region (217.24 million, 95% CI = 168.22-275.26). Across WHO regions, the prevalence of mVAD was the highest in AFR, with almost one-third of children with mVAD (30.12%, 95% CI = 19.77-43.11). However, the most mVAD cases were noted in SEAR (171.10 million, 95% CI = 130.81-219.60).

**Table 2:** The estimated prevalence and number of cases of marginal vitamin A deficiency (mVAD) among children residing in low- and middle-income countries (LMICs) in 2019, by age group

Region	0-5 years		6-12 years		13-18 years		0-18 years	
	**Prevalence (%)**	**Cases (million)**	**Prevalence (%)**	**Cases (million)**	**Prevalence (%)**	**Cases (million)**	**Prevalence (%)**	**Cases (million)**
**LMIC overall**	28.22 (20.00-38.24)	211.94 (150.25-287.17)	24.28 (17.01-33.51)	203.95 (142.88-281.47)	20.76 (14.16-29.50)	140.24 (95.69-199.30)	24.54 (17.15-33.88)	556.13 (388.83-767.94)
**Development region**								
High-middle SDI	18.50 (10.74-29.74)	10.57 (6.13-16.99)	15.91 (9.07-26.24)	10.66 (6.08-17.58)	13.55 (7.54-23.07)	7.16 (3.98-12.18)	16.04 (9.15-26.42)	28.38 (16.19-46.75)
Middle SDI	22.57 (16.01-30.76)	58.08 (41.21-79.17)	19.44 (13.58-26.99)	56.80 (39.68-78.88)	16.55 (11.22-23.71)	39.63 (26.86-56.78)	19.58 (13.66-27.23)	154.52 (107.75-214.83)
Low-middle SDI	29.84 (23.48-37.16)	79.85 (62.84-99.45)	25.90 (20.13-32.71)	80.07 (62.23-101.13)	22.33 (16.81-29.09)	57.33 (43.16-74.68)	26.06 (20.18-33.02)	217.24 (168.22-275.26)
Low SDI	37.55 (23.72-54.19)	63.44 (40.07-91.56)	32.90 (20.35-48.91)	56.42 (34.89-83.88)	28.53 (17.13-43.96)	36.13 (21.69-55.66)	33.40 (20.70-49.48)	155.99 (96.66-231.11)
**WHO region**								
AFR	33.85 (22.64-47.31)	68.52 (45.83-95.77)	29.56 (19.40-42.42)	60.19 (39.50-86.38)	25.66 (16.29-38.21)	37.17 (23.59-55.33)	30.12 (19.77-43.11)	165.89 (108.92-237.49)
AMR	20.63 (15.17-27.29)	12.85 (9.45-16.99)	17.77 (12.88-23.91)	12.96 (9.39-17.44)	15.18 (10.67-21.07)	9.72 (6.83-13.49)	17.83 (12.89-24.05)	35.52 (25.68-47.92)
EMR	29.36 (20.37-40.23)	29.80 (20.67-40.82)	25.54 (17.41-35.81)	26.95 (18.37-37.80)	22.10 (14.62-32.03)	17.56 (11.61-25.45)	25.94 (17.68-36.33)	74.30 (50.66-104.07)
EUR	20.04 (12.22-31.21)	9.49 (5.79-14.78)	17.08 (10.16-27.42)	9.37 (5.57-15.04)	14.44 (8.30-24.07)	6.05 (3.48-10.09)	17.28 (10.30-27.69)	24.91 (14.84-39.91)
SEAR	29.45 (22.90-37.11)	60.00 (46.66-75.61)	25.52 (19.59-32.62)	63.22 (48.54-80.82)	21.97 (16.33-28.98)	47.89 (35.61-63.18)	25.56 (19.54-32.80)	171.10 (130.81-219.60)
WPR	23.38 (16.34-32.29)	31.29 (21.85-43.19)	20.14 (13.85-28.35)	31.26 (21.50-44.00)	17.15 (11.43-24.93)	21.85 (14.57-31.76)	20.27 (13.91-28.57)	84.40 (57.92-118.95)

mVAD – marginal vitamin A deficiency, LMICs – low- and middle-income countries, SDI – socio-demographic index, WHO – The World Health Organization, AFR – African Region, AMR – Region of the Americas, SEAR – South-East Asia Region, EUR – European region, EMR – Eastern Mediterranean region, WPR – Western Pacific Region

**Figure 2:**
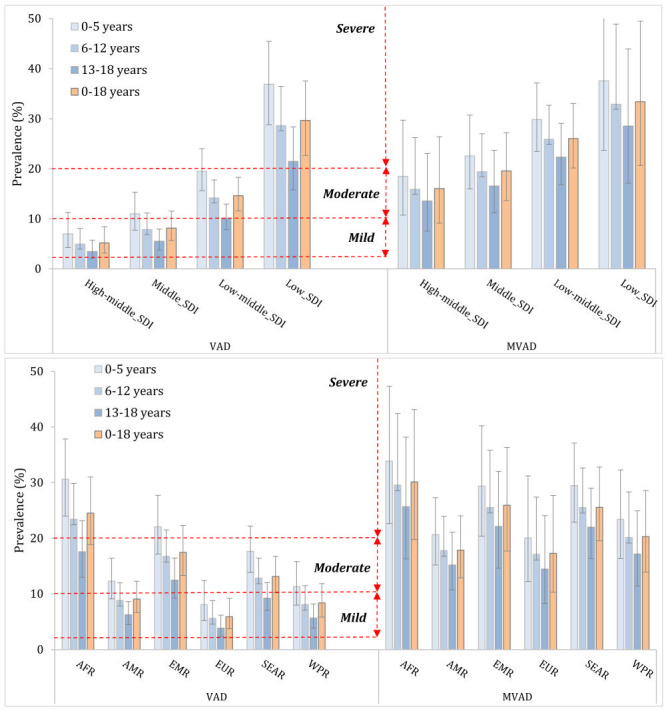
Classification of regions by degree of public health significance of vitamin A deficiency. SDI – socio-demographic index, AFR – African Region, AMR – Region of the Americas, EMR – Eastern Mediterranean Region, EUR – European Region, VAD – vitamin A deficiency, mVAD – marginal vitamin A deficiency

As shown in **Figure 2**, low-SDI region was classified as having a severe public health problem based on biochemical VAD in children (0-18 years). In terms of age subgroups, the public health significance of VAD for children aged 0-5 years and 6-12 years were severe in low-SDI region, while the problem was moderate for children aged 13-18 years. Geographically, the public health significance of VAD was severe for children aged 0-5 years and 6-12 years in AFR. For adolescents aged 13-18 years in AFR, children aged 0-5 years and 6-12 years in EMR and SEAR, the public health significance of VAD was moderate. When taking children aged 0-18 years as a whole, AFR, EMR and SEAR had a moderate public health problem of VAD.

**Figure 3:**
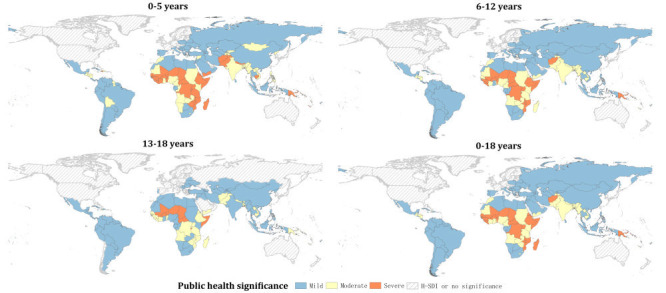
Classification of countries by degree of public health significance of vitamin A deficiency (VAD). High- socio-demographic index (H-SDI), including countries with SDI ≥0.805; a lower confidence interval of VAD prevalence of ≥2% but <10% as mild; ≥10% but <20% as moderate; and ≥20% as severe areas of public health significance.

Across LMICs (**Figure 3**), 68 countries were classified as having a moderate to severe public health problem of VAD. For children aged 0-5 years, 84 countries were with a moderate to severe public health problem of VAD. The number of countries with a moderate to severe public health problem of VAD was 65 among children aged 6-12 years, with this reducing to 46 among adolescents aged 13-18 years. National prevalence of VAD and its public health significance are listed in the Table S7 in the **Online Supplementary Document**.

## DISCUSSION

This study included high quality data from 40 LMICs, covering approximately 300 000 children and adolescents. The prevalence of VAD and mVAD in 2019 was estimated to be 14.73% (95% CI = 11.16-19.14) and 24.54% (95% CI = 17.15-33.88), accounting for more than 330 million and 550 million children in LMICs, respectively. The prevalence estimates of VAD and mVAD were both the highest among children under five years and gradually decreased with increasing age. Our findings suggest that the level of development, which serves as a proxy for access to essential nutrition and health resources, is a significant determinant of VAD prevalence, with significantly higher prevalence of VAD observed in the low SDI region. Finally, over 40% of LMICs were considered of significant public health concern of VAD.

In the GBD 2019 study, which utilised 46 data sources, a global age standardised prevalence of VAD was estimated to be 7.0%, accounting for 23.1 million cases. The highest prevalence rates were indicated in Central and Eastern sub-Saharan Africa, with rates of 25.9% and 23.5%, respectively [8,19,20]. It is important to note that these significantly lower estimates in GBD 2019 may have been influenced by various factors, such as data availability, model assumptions, analytical methods, and especially the case definitions employed, which were based on a combination of low serum retinol concentration (<0.7 μmol / l) and blindness and vision loss due to VAD.

The results of our study support previous global research that has reported considerably higher prevalence of VAD in AFR and SEAR. In 2005, the WHO estimated that 33.3% of preschool-age children, equivalent to 190 million children, had VAD, with a majority of cases in SEAR (92 million, 50%) and AFR (56 million, 44%) alone [4]. A more recent study by Stevens et al. in 2015, utilising data from 83 countries, estimated a global VAD prevalence of 29%, with the highest rates observed in sub-Saharan Africa (48%) and South Asia (44%) [1]. Our findings for children aged five years or younger are consistent with these estimates, with a global prevalence of 19.53% and 146.68 million cases in LMICs. Our results also suggest that the EMR may be facing an increasing burden of VAD, as we estimated a higher VAD prevalence (22.07%) among children aged 0-5 years in this region compared to the SEAR (17.62%). This pattern is also evident when considering the total 0-18 years age group, with a higher prevalence of VAD in EMR (17.43%) compared to SEAR (13.11%), although the latter had a higher absolute number of child cases. This previously undiscovered trend in the EMR requires further investigation.

Indeed, previous studies have reported a decrease in the prevalence of VAD, largely attributed to successful interventions in HICs. However, our estimates for LMICs indicate a more gradual decrease, with a VAD prevalence of 14.73% and a mVAD prevalence of 24.54% in 2019. This decrease may be due to persistent gaps in LMICs, which could obscure the overall trend in the global scene. Our results also highlight the disproportionate burden of VAD in low-income regions of LMICs, with a 5-fold difference in VAD prevalence compared to high-middle income regions. A typical example is the prevalence of VAD among children and adolescents in China in 2016 estimated at 0.96% [19,21], whereas a similar micronutrient surveillance in Bangladesh estimated a VAD prevalence of 76.8% among preschool children [22]. Monitoring of vitamin A status in vulnerable and underprivileged populations in LMICs would therefore be important, with consumption of low-cost diet rich in vitamin A encouraged.

Data gaps across LMICs remain a major challenge in the estimation of the prevalence of VAD. This study was deemed necessary as its findings have direct or indirect potential to inform policy making, formulise priority settings, and target maternal and child nutrition interventions [6,23]. The strengths of this study are still related to its comprehensive modelling with estimates across different child age-groups (0-5, 6-12 and 13-18 years), development regions and geographic areas. We have also, to the best of our knowledge, provided regional and national level estimates for mVAD, which is an important contribution to the understanding of global vitamin A epidemiology, perhaps vital for early identification of VAD prone spots and future research. Besides, providing estimates of mVAD which will be vital for early identification of vitamin A deficient areas, we have also provided estimates for ages 5-18 years, which complements, and also an improvement on, previous estimates mostly based on children under five years.

However, several limitations should also be noted. First, data from many countries in AFR, SEAR, and EMR, where most areas of public health significance are located, are missing. A study published in 2017 showed that two-thirds of countries with ongoing VAS programmes lacked data on VAD to drive appropriate response, or that data were just too old to be relevant when available [2]. This raises some uncertainties in our estimation of country level prevalence rates which should also guide interpretation of our results. In future research, developing standardised data collection tools and protocols and leveraging technological advancements such as mobile health applications and digital platforms for data collection are required. Additionally, efforts should be made to ensure transparency and collaboration among researchers, which would facilitate data sharing, harmonisation, and ultimately contribute to more accurate estimates of VAD burden globally. Second, Vitamin A is stored in the liver and transported to the end organs by retinol binding protein (RBP) [24]. Therefore, circulating concentrations of RBP, the carrier protein for serum (plasma) retinol, are also considered a biological indicator of VAD in some large investigations [25]. While, in order to reduce to a large extent the heterogeneity caused by case definitions, serum (plasma) retinol concentrations were used uniformly in this study for the assessment of vitamin A status. Notably, a limitation of using serum (plasma) retinol concentration as an indicator of vitamin A status is that it is temporarily decreased by acute infections and inflammatory processes, which may overestimate VAD in some populations [26]. Not controlling for inflammation in a population when assessing nutritional status may lead to inaccurate prevalence estimates. However, the majority of surveys do not use indicators of inflammation status when assessing retinol. Moreover, due to limited data, we could not incorporate data on determinants or risk factors for VAD into our model. This could have contributed to a more robust model particularly for the distribution of the country level estimates. To improve the accuracy and robustness of VAD prevalence estimation, future studies should consider incorporating the potential risk factors into the analytical models, like inflammation status. It also has implications for addressing VAD effectively would also imply identifying and managing those at risk. Although SDI was not statistically significant for mVAD, this was added to the regression models of mVAD prevalence estimation for reasons already described, and to keep consistency between models.

Continued and intensified effort to address VAD in AFR and SEAR most vulnerable groups is still encouraged given the results from this study. As at 2017, approximately 100 countries globally were implementing VAS programmes, targeting over 270 million children aged 6-59 months [2]. Despite some ongoing debate, these programmes are believed to contribute to reducing under five deaths, particularly from measles and diarrhoea in LMICs. However, the programs do not address inadequate vitamin A intake among children [27,28]. Countries like Nigeria, Zambia and Malaysia have more than 80% coverage of VAS programs, yet with relatively high VAD prevalence, suggesting a need for a more encompassing response [29-31].

The need for up-to-date information on vitamin A in many LMICs remains a priority in the response to VAD [31]. Accurate representation of VAD epidemiology is crucial and can be achieved through further research in under-represented areas of public health significance. This study would present valuable opportunity to identify child populations and community clusters at risks that can in turn guide evidence-based advocacy while also contributing to designing and delivering effective interventions [1]. With a good understanding of population needs, experts have recommended combination of interventions that include food-based approaches like fortification and biofortification of diets and dietary diversification, and providing relevant education on nutrition and prevention and control of common childhood infections [5].

## CONCLUSIONS

In summary, although sub-Saharan Africa and SEAR remain most affected by VAD, the prevalence in the EMR may be gradually increasing. Development level in many LMICs is also a key determinant with a considerably higher burden in low SDI region. Given data limitations, further studies in many LMICs are recommended for improved understanding of VAD epidemiology necessary to guide relevant policy shift, reforms and programme needs.

## Additional material


Online Supplementary Document

## References

[1] Stevens GA Bennett JE Hennocq Q Lu Y De-Regil LM Rogers L Trends and mortality effects of vitamin A deficiency in children in 138 low-income and middle-income countries between 1991 and 2013: a pooled analysis of population-based surveys. Lancet Glob Health. 2015;3:e528-36. 10.1016/S2214-109X(15)00039-X 26275329

[2] Wirth JP Petry N Tanumihardjo SA Rogers LM McLean E Greig A Vitamin A supplementation programs and country-level evidence of vitamin A deficiency. Nutrients. 2017;9:190. 10.3390/nu9030190 28245571 PMC5372853

[3] Bailey RL West KP Jr Black RE The epidemiology of global micronutrient deficiencies. Ann Nutr Metab. 2015;66:22-33. 10.1159/000371618 26045325

[4] World Health Organization. Global prevalence of vitamin A deficiency in populations at risk 1995-2005: WHO global database on vitamin A deficiency. Geneva: World Health Organization; 2009.

[5] Fawzi WW Wang D When should universal distribution of periodic high-dose vitamin A to children cease? Am J Clin Nutr. 2021;113:769-71. 10.1093/ajcn/nqaa428 33751042

[6] Ahmed T Hossain M Sanin KI Global burden of maternal and child undernutrition and micronutrient deficiencies. Ann Nutr Metab. 2012;61:8-17. 10.1159/000345165 23343943

[7] Han X Ding S Lu J Li Y Global, regional, and national burdens of common micronutrient deficiencies from 1990 to 2019: A secondary trend analysis based on the Global Burden of Disease 2019 study. EClinicalMedicine. 2022;44:101299. 10.1016/j.eclinm.2022.101299 35198923 PMC8850322

[8] Zhao T Liu S Zhang R Zhao Z Yu H Pu L Global burden of vitamin A deficiency in 204 countries and territories from 1990-2019. Nutrients. 2022;14:950. 10.3390/nu14050950 35267925 PMC8912822

[9] Hess SY McLain AC Frongillo EA Afshin A Kassebaum NJ Osendarp SJM Challenges for estimating the global prevalence of micronutrient deficiencies and related disease burden: a case study of the global burden of disease study. Curr Dev Nutr. 2021;5:nzab141. 10.1093/cdn/nzab141 34993390 PMC8728001

[10] Page MJ McKenzie JE Bossuyt PM Boutron I Hoffmann TC Mulrow CD The PRISMA 2020 statement: an updated guideline for reporting systematic reviews. BMJ. 2021;372:n71. 10.1136/bmj.n71 33782057 PMC8005924

[11] Stroup DF Berlin JA Morton SC Olkin I Williamson GD Rennie D Meta-analysis of observational studies in epidemiology: a proposal for reporting. Meta-analysis Of Observational Studies in Epidemiology (MOOSE) group. JAMA. 2000;283:2008-12. 10.1001/jama.283.15.2008 10789670

[12] Song P Wang J Wei W Chang X Wang M An L The prevalence of vitamin A deficiency in Chinese children: a systematic review and Bayesian meta-analysis. Nutrients. 2017;9:1285. 10.3390/nu9121285 29186832 PMC5748736

[13] Global Burden of Disease Collaborative Network. Global Burden of Disease Study 2019 (GBD 2019) Socio-Demographic Index (SDI) 1950-2019. Seattle, United States of America: Institute for Health Metrics and Evaluation (IHME); 2020.

[14] National Institution of Health. U.S Department of Health and Human Services (2015) Quality Assessment Tool. Available: https://www.nhlbi.nih.gov/health-topics/study-quality-assessment-tools. Accessed: 12 April 2022.

[15] Hox JJ, Moerbeek M, Van de Schoot R. Multilevel analysis: Techniques and applications. Routledge; 2017.

[16] United Nations, Department of Economic and Social Affairs, Population Division. World Population Prospects, the 2017 Revision. Available: https://esa.un.org/unpd/wpp/. Accessed: 13 April 2022.

[17] Song P Xia W Zhu Y Wang M Chang X Jin S Prevalence of carotid atherosclerosis and carotid plaque in Chinese adults: A systematic review and meta-regression analysis. Atherosclerosis. 2018;276:67-73. 10.1016/j.atherosclerosis.2018.07.020 30036743

[18] Song P Rudan D Zhu Y Fowkes FJ Rahimi K Fowkes FGR Global, regional, and national prevalence and risk factors for peripheral artery disease in 2015: an updated systematic review and analysis. Lancet Glob Health. 2019;7:e1020-30. 10.1016/S2214-109X(19)30255-4 31303293

[19] Wang R Zhang H Hu YC Chen J Yang Z Zhao L Serum vitamin A nutritional status of children and adolescents aged 6-17 years - China, 2016-2017. China CDC Wkly. 2021;3:189-92. 10.46234/ccdcw2021.057 34595041 PMC8393032

[20] GBD 2019 Diseases and Injuries Collaborators Global burden of 369 diseases and injuries in 204 countries and territories, 1990-2019: a systematic analysis for the Global Burden of Disease Study 2019. Lancet. 2020;396:1204-22. 10.1016/S0140-6736(20)30925-9 33069326 PMC7567026

[21] Xu Y Shan Y Lin X Miao Q Lou L Wang Y Global patterns in vision loss burden due to vitamin A deficiency from 1990 to 2017. Public Health Nutr. 2021;24:5786-94. 10.1017/S1368980021001324 33775269 PMC10195433

[22] Fiedler JL Lividini K Bermudez OI Estimating the impact of vitamin A-fortified vegetable oil in Bangladesh in the absence of dietary assessment data. Public Health Nutr. 2015;18:414-20. 10.1017/S1368980014000640 24762782 PMC10271769

[23] Arora NK Mohapatra A Gopalan HS Wazny K Thavaraj V Rasaily R Setting research priorities for maternal, newborn, child health and nutrition in India by engaging experts from 256 indigenous institutions contributing over 4000 research ideas: a CHNRI exercise by ICMR and INCLEN. J Glob Health. 2017;7:011003. 10.7189/jogh.07.011003 28686749 PMC5481897

[24] Likoswe BH Joy EJM Sandalinas F Filteau S Maleta K Phuka JC Re-defining the population-specific cut-off mark for vitamin A deficiency in pre-school children of Malawi. Nutrients. 2021;13:849. 10.3390/nu13030849 33807563 PMC8000145

[25] Craft NE, Furr HC. Methods for assessment of vitamin A (retinoids) and carotenoids. Laboratory assessment of vitamin status: Elsevier; 2019.

[26] Rubin LP Ross AC Stephensen CB Bohn T Tanumihardjo SA Metabolic effects of inflammation on vitamin A and carotenoids in humans and animal models. Adv Nutr. 2017;8:197-212. 10.3945/an.116.014167 28298266 PMC5347109

[27] Mason JB Benn CS Sachdev H West KP Jr Palmer AC Sommer A Should universal distribution of high dose vitamin A to children cease? BMJ. 2018;360:k927. 10.1136/bmj.k927 29496673

[28] Klemm RD Palmer AC Greig A Engle-Stone R Dalmiya N A changing landscape for vitamin A programs: implications for optimal intervention packages, program monitoring, and safety. Food Nutr Bull. 2016;37:S75-86. 10.1177/0379572116630481 27004480

[29] Fiedler JL Lividini K Kabaghe G Zulu R Tehinse J Bermudez OI Assessing Zambia’s industrial fortification options: Getting beyond changes in prevalence and cost-effectiveness. Food Nutr Bull. 2013;34:501-19. 10.1177/156482651303400413 24605698

[30] Tan PY Mohd Johari SN Teng K-T Loganathan R Lee SC Ngui R High prevalence of malnutrition and vitamin A deficiency among schoolchildren of rural areas in Malaysia using a multi-school assessment approach. Br J Nutr. 2023;129:454-67. 10.1017/S0007114522001398 35506400

[31] Mason JB Sanders D Greiner T Shrimpton R Yukich J Vitamin A deficiency: policy implications of estimates of trends and mortality in children. Lancet Glob Health. 2016;4:e21. 10.1016/S2214-109X(15)00246-6 26718802

