# Supplementary material for: The prevalence of vitamin A deficiency and its public health significance in children in low- and middle-income countries: A systematic review and modelling analysis
Source: J Glob Health. 2023 Aug 11;13:04084. doi: 10.7189/jogh.13.04084 (PMC10416138; doi:10.7189/jogh.13.04084)
Supplement: Online Supplementary Document [file jogh-13-04084-s001.pdf]

ONLINE SUPPLEMENTARY DOCUMENT

Title: The Prevalence of Vitamin A Deficiency and Its Public Health Significance in Children in Low- and Middle-Income Countries: A Systematic Review and Modelling Analysis

Authors: Peige Song*, Davies Adeloye*, Shuting Li, Dong Zhao, Xinxin Ye, Qing Pan, Yiwen Qiu, Ronghua Zhang, Igor Rudan; Global Health Epidemiology Research Group (GHERG)

Table S1. Search strategy to identify studies reporting the prevalence of VAD or mVAD

Table S2. The time lag between investigation and publication in the included articles (n=116)

Table S3. Quality assessment scale

for rating the risk of bias

Table S4. Age-adjusted meta-regression models of cluster-level factors related to the prevalence of VAD and mVAD (logit form)

Table S5. Detailed characteristics of the included articles (n=116)

Table S6. Quality scores for assessing the risk of bias in the included articles (n=116)

Table S7. National prevalence of VAD and public health significance in 2019, by age group

Figure S1. Contributing data sources across world regions

Figure S2. The relations of age and prevalence of VAD and mVAD, by SDI

eMethods. Detailed process of the estimation of the prevalence of VAD or mVAD

References

This supplementary material has been provided by the authors to give readers additional information about their work.

Table S1. Search strategy to identify studies reporting the prevalence of VAD or mVAD

Database	Access date	Search terms
**PubMed**	5th Oct, 2022	('vitamin a'[Title/Abstract] OR retinol[Title/Abstract] OR retinal[Title/Abstract] OR 'aquasol a' [Title/Abstract] OR 'night blindness'[Title/Abstract] OR 'Bitot's spots'[Title/Abstract] OR xerophthalmia[Title/Abstract]) AND (infan* OR child* OR teen* OR adolescen* OR pediatric* OR paediatric*) AND (prevalence[Title/Abstract] OR epidemiolog*[Title/Abstract]) AND ("2000/01/01"[Date - Publication] : "3000"[Date - Publication])
**MEDLINE (1950-)**	5th Oct, 2022	1 exp Vitamin A Deficiency/<br>2 ('vitamin a' or retinol or retinal or 'aquasol a' or 'night blindness' or 'Bitot spots' or xerophthalmia).ab,ti.<br>3 (infan* or child* or teen* or adolescen* or pediatric* or paediatric*).ab,ti.<br>4 (prevalen* or epidemiolog*).ab,ti.<br>5 1 or 2<br>6 3 or 4<br>7 5 and 6<br>8 limit 7 to (humans and yr="2000 -Current" and medline)
**EMBASE (1980-)**	5th Oct, 2022	1 ('vitamin a deficiency':ab,ti OR 'vitamin a':ab,ti OR retinol:ab,ti OR retinal:ab,ti OR 'aquasol a':ab,ti OR 'night blindness':ab,ti OR 'Bitot's spots':ab,ti OR xerophthalmia:ab,ti)<br>2 (infan*:ab,ti OR child*:ab,ti OR teen*:ab,ti OR adolescen*:ab,ti OR pediatric*:ab,ti OR paediatric*:ab,ti)<br>3 (prevalen*:ab,ti or epidemiolog*:ab,ti)<br>4 #1 AND #2 AND #3<br>6 #4 AND (2000:py OR 2001:py OR 2002:py OR 2003:py OR 2004:py OR 2005:py OR 2006:py OR 2007:py OR 2008:py OR 2009:py OR 200:py OR 2011:py OR 2012:py OR 2013:py OR 204:py OR 2015:py OR 2016:py OR 2017:py OR 2018:py OR 2019:py OR 2020:py OR 2021:py OR 2022:py OR) AND [embase]/lim AND 'human'/de AND 'article'/it

Table S2. The time lag between investigation and publication in the included articles (n=116)

Study ID	Author(s)	Year of publication	Year of investigation	Time-lag (year)
V1	Disalvo L, et al.[1]	2019	2011	8
V2	Onoja US, et al.[2]	2019	2017	2
V3	Hanson C, et al.[3]	2018	2018	0
V4	Ayogu R, et al.[4]	2018	2018	0
V5	Pouraram H et al.[5]	2018	2013	5
V6	Abolurin OO, et al.[6]	2018	2016	2
V7	Lima DB, et al.[7]	2018	2006	12
V8	Chyne D, et al.[8]	2017	2014	3
V9	Longvah T, et al.[9]	2017	2013	4
V10	Alaofè H, et al.[10]	2017	2014	3
V11	Rahman S, et al.[11]	2017	2012	5
V12	Alouache A, et al.[12]	2017	2009	8
V13	Ayogu RN, et al.[13]	2016	NA	NA
V14	Wei X ,et al.[14]	2016	NA	NA
V15	Villalpando, et al.[15]	2015	2012	3
V16	Talsma EF, et al.[16]	2015	2010	5
V17	Marasinghe E, et al.[17]	2015	NA	NA
V18	Sandjaja, et al.[18]	2015	2011	4
V19	Faber M, et al.[19]	2015	2011	4
V20	Konstantyner T, et al.[20]	2014	2006	8
V21	Herrador Z, et al.[21]	2014	2009	5
V22	Ribeiro-Silva RC, et al.[22]	2014	2009	5
V23	Martínez-Torres J, et al.[23]	2014	2010	4
V24	Dong C, et al.[24]	2014	2009	5
V25	Rohner F, et al.[25]	2014	2007	7
V26	Rojroongwasinkul N, et al.[26]	2013	2011	2
V27	Poh BK, et al.[27]	2013	2011	2
V28	Queiroz D, et al.[28]	2013	2007	6
V29	Laillou A, et al.[29]	2012	2010	2
V30	Hotz C, et al.[30]	2012	2009	3
V31	Laxmaiah A, et al.[31]	2012	2004	8
V32	Van Stuijvenberg ME, et al.[32]	2012	2008	4
V33	Engle-Stone R, et al.[33]	2011	NA	NA
V34	Temple VJ, et al.[34]	2011	2008	3
V35	Arlappa N, et al.[35]	2011	2003	8
V36	Arlappa N, et al.[36]	2011	2003	8
V37	Fares S, et al.[37]	2011	NA	NA
V38	Durán P, et al.[38]	2011	2005	6
V39	Dillon D, et al.[39]	2010	2001	9
V40	Custodio VI, et al.[40]	2009	2004	5
V41	Khatib IMD, et al.[41]	2009	2004	5

Study ID	Author(s)	Year of publication	Year of investigation	Time-lag (year)
V42	Tatala SR, et al.[42]	2008	1996	12
V43	Jiang JX, et al.[43]	2008	2000	8
V44	Calis JCJ, et al.[44]	2008	2003	5
V45	Al-Mekhlafi MH, et al.[45]	2007	2004	3
V46	Yang R, et al.[46]	2007	2006	1
V47	Khan NC, et al.[47]	2007	NA	NA
V48	Jiang J, et al.[48]	2006	2000	6
V49	Hix J, et al.[49]	2006	2001	5
V50	Maziya-Dixon B.B, et al.[50]	2006	2001	5
V51	Thurlow R.A, et al.[51]	2005	2002	3
V52	Santos M.A, et al.[52]	2005	NA	NA
V53	Nasri I, et al.[53]	2004	NA	NA
V54	Coles C.L, et al.[54]	2004	1996	8
V55	Gamble M.V, et al.[55]	2004	1994	10
V56	Yamamura C.M, et al.[56]	2004	1993	11
V57	Villalpando S, et al.[57]	2003	1999	4
V58	Adelekan D.A, et al.[58]	2003	1997	6
V59	Oso O.O, et al.[59]	2003	1993	10
V60	Palafox N.A, et al.[60]	2003	1995	8
V61	Tan Z, et al.[61]	2002	2000	2
V62	Schémann J.F, et al.[62]	2002	1997	5
V63	Swami H.M, et al.[63]	2001	2000	1
V64	Centers for Disease Control Prevention[64]	2001	2000	1
V65	Kassaye T, et al.[65]	2001	1997	4
V66	Centers for Disease Control Prevention[66]	1999	1999	0
V67	Ferraz I.S, et al.[67]	2000	1997	3
V68	Li X.C, et al.[68]	2020	2009	11
V69	Zhang Z, et al.[69]	2018	2016	2
V70	Gubert M.B, et al.[70]	2016	2006	10
V71	Jin C, et al.[71]	2016	2015	1
V72	Pajuelo J, et al.[72]	2015	2009	6
V73	De Moura F.F, et al.[73]	2015	2011	4
V74	Kurihayashi A.Y, et al.[74]	2015	2013	2
V75	Augusto R.A, et al.[75]	2015	2007	8
V76	Peng R, et al.[76]	2014	2012	2
V77	de Paula W.K, et al.[77]	2014	2006	8
V78	Rohner F, et al.[78]	2013	2008	5
V79	Miglioli T.C, et al.[79]	2013	2006	7
V80	Fan P, et al.[80]	2012	2009	3
V81	Garcia M.T, et al.[81]	2011	2007	4
V82	Midyat L, et al.[82]	2011	2006	5

Study ID	Author(s)	Year of publication	Year of investigation	Time-lag (year)
V83	Azevedo M.M, et al.[83]	2010	2007	3
V84	Demissie T, et al.[84]	2010	2006	4
V85	Khatib I.M., et al.[85]	2009	NA	NA
V86	Durán P, et al.[86]	2009	2005	4
V87	Maslova E, et al.[87]	2009	2006	3
V88	Leal J.Y, et al.[88]	2007	2007	0
V89	Graebner I.T, et al.[89]	2007	2003	4
V90	Poveda E, et al.[90]	2007	2007	0
V91	de Souza V.D.S.L, et al.[91]	2007	2002	5
V92	Zhang Y, et al.[92]	2007	2002	5
V93	Faruque A.S, et al.[93]	2006	1998	8
V94	Paiva A.A, et al.[94]	2006	NA	NA
V95	Li J.H, et al.[95]	2006	2002	4
V96	Ahmed F, et al.[96]	2006	2000	6
V97	Ferraz I.S, et al.[97]	2005	2000	5
V98	Monge-Rojas R, et al.[98]	2005	NA	NA
V99	Sibeko L.N, et al.[99]	2004	2000	4
V100	Martins M.C, et al.[100]	2004	1998	6
V101	Castejon H.V, et al.[101]	2004	2004	0
V102	Mi J, et al.[102]	2003	2001	2
V103	Lin L, et al.[103]	2002	2000	2
V104	Amaya-Castellanos D, et al.[104]	2002	NA	NA
V105	Oelofse A, et al.[105]	2002	1998	4
V106	Jinabhai C.C, et al.[106]	2001	NA	NA
V107	Dijkhuizen M.A, et al.[107]	2001	1996	5
V108	Albalak R, et al.[108]	2000	1996	4
V109	Gowele VF, et al.[109]	2021	2016	5
V110	Reddy GB, et al.[110]	2022	2017	5
V111	De Castro IRR, et al.[111]	2021	2014	7
V112	Reddy GB, et al.[112]	2021	2017	4
V113	Tian T, et al.[113]	2022	2017	5
V114	Janmohamed A, et al.[114]	2020	2017	3
V115	Shan X, et al.[115]	2021	2016	5
V116	Ernawati F, et al.[116]	2021	2011	10

Note: The average time-lag between investigation and publication was 4.84 based on 103 articles with available data.

Table S3. Quality assessment scale for rating the risk of bias

Bias type	Low risk (score=2)	Moderate risk (score=1)	High risk (score=0)
Selection (sample population)	1) Sample from the general population, not a select group;<br>2) Consecutive unselected population;<br>3) Rationale for case and control selection explained.	1) Sample selected from large population but selection criteria not defined;<br>2) Sample selection ambiguous but may be representative;<br>3) Rationale for cases and controls not explained;<br>4) Eligibility criteria not explained;<br>5) Analysis to adjust for sampling strategy bias.	1) Highly select population making it difficult to generalise finding;<br>2) Sample selection ambiguous and sample unlikely to be representative.
Selection (sample size)	1) Sample size calculation performed and adequate.	1) Sample size calculation performed and reasons for not meeting sample size given;<br>2) Sample size calculation not performed but all eligible persons studied.	1) Sample size estimation unclear or only sub-sample studied.
Selection (participation rate)	1) High response rate (>85%).	1) Moderate response rate (70-85%).	1) Low response rate (<70%);<br>2) Response rate not reported.
Performance bias (outcome assessment)	1) Diagnosis using consistent criteria and direct examination.	1) Assessment from administrative database or register;<br>2) Assessment from hospital record or interviewer.	1) Assessment from non-validated data or generic estimate from the overall population.
Performance bias (analytical methods to control for bias)	1) Analysis appropriate for the type of sample (subgroup analysis/regression etc.).	1) Analysis does not account for common adjustment.	1) Data confusing.

Table S4. Age-adjusted meta-regression models of cluster-level factors related to the prevalence of VAD and mVAD (logit form)

Moderator	Number of articles	Number of data points	β	95 % CI		*P* value
			**VAD**			
Age (crude)	115	257	-0.0579	-0.0654	-0.0505	<0.0001
Proportion of girls	90	192	-0.0467	-0.0882	-0.0053	0.027
Sex						
Girl	29	37	Reference			
Boy	30	38	0.0469	0.0055	0.0884	0.0264
Setting						
Rural	33	77	Reference			
Urban	23	46	-0.3359	-0.3992	-0.2727	<0.0001
SDI	115	257	-4.8503	-6.6723	-3.0284	<0.0001
Development Region						
High-middle SDI	64	152	Reference			
Low-middle and low SDI	51	105	0.713	0.1378	1.2883	0.0151
WHO region						
WPR	30	78	Reference			
AFR	27	54	1.1659	0.3345	1.9973	0.006
AMR	31	68	0.187	-0.703	1.0771	0.6805
SEAR	20	45	0.4193	-0.5095	1.348	0.3762
EUR	2	2	0.1082	-1.7339	1.9502	0.9084
EMR	5	10	0.2178	-1.0524	1.488	0.7368
Investigation period						
Before 2000	19	41	Reference			
2000-2009	61	148	-1.1451	-1.7372	-0.5529	0.0002
2010 and later	35	68	-1.4466	-2.0789	-0.8143	<0.0001
Test						
HPLC	99	218	Reference			
Other/mixed	16	39	-0.2173	-0.8474	0.4128	0.4991
Investigation site						
Community-based	72	175	Reference			
Facility-based	43	82	-0.0968	-0.5754	0.3819	0.6919
			**mVAD**			
Age (crude)	40	86	-0.0302	-0.0444	-0.0159	<0.0001
Proportion of girls	35	84	-0.1056	-0.2251	0.0139	0.0833
Sex						
Girl	10	12	Reference			
Boy	11	13	0.1044	-0.0149	0.2236	0.0863
Setting						
Rural	7	24	Reference			
Urban	7	13	-1.0478	-1.1395	-0.956	<0.0001
SDI	40	86	-1.5359	-4.4351	1.3633	0.2991
WB Region						
High-middle SDI	29	68	Reference			
Low-middle and low SDI	11	18	0.3027	-0.428	1.0333	0.4168
WHO region						
WPR	17	51	Reference			
AFR	3	3	-0.5165	-2.0406	1.0077	0.5066
AMR	12	20	-0.4664	-1.5413	0.6085	0.395
SEAR	5	8	-0.6579	-1.9952	0.6793	0.3349
EUR	1	1	0.2284	-2.1679	2.6248	0.8518
EMR	2	3	-0.0069	-1.7734	1.7597	0.9939
Investigation period						

Moderator	Number of articles	Number of data points	β	95 % CI		*P* value
Before 2000	5	10	Reference			
2000-2009	22	60	-0.4329	-1.461	0.5953	0.4093
2010 and later	13	16	-0.3193	-1.41	0.7714	0.5661
Test						
HPLC	35	76	Reference			
Other/mixed	5	10	0.5246	-0.4143	1.4636	0.2735
Investigation site						
Community-based	22	58	Reference			
Facility-based	18	28	0.0034	-0.6385	0.6454	0.9916

Note: VAD= Vitamin A Deficiency; mVAD= Marginal Vitamin A Deficiency; SDI= Socio-demographic Index; AFR=African Region. AMR=Region of the Americas. EMR=Eastern Mediterranean Region. EUR=European Region; HPLC=High-performance liquid chromatography.

Table S5. Detailed characteristics of the included articles (n=116)

ID	Author	Year Published	Country	WHO region	WB region	SDI	Setting	Study year	Survey base	Test	Tested sample	No. of VAD cases	No. of mVAD cases	Age range	Age-specific estimate	Sex-specific estimate
V1	Disalvo L, et al.[1]	2019	Argentina	AMR	UMIC	0.67	Urban	2011	Community-based	UFLC	624	152	358	1-6y	Yes	Yes
V2	Onoja US, et al.[2]	2019	Nigeria	AFR	LMIC	0.503	Both	2017	School-based	NS	174	69	NS	10-19y	Yes	Yes
V3	Hanson C, et al.[3]	2018	Nigeria	AFR	LMIC	0.51	Mixed	2018	Health check	HPLC	74	61	12	0-7d	No	No
V4	Ayogu R, et al.[4]	2018	Nigeria	AFR	LMIC	0.51	Rural	2018	School-based	HPLC	90	46	NS	6-15y	No	No
V5	Pouraram H et al.[5]	2018	Iran	EMR	UMIC	0.64	Both	2013	Community-based	HPLC	4261	790	NS	15-23m	No	Yes
V6	Abolurin OO, et al.[6]	2018	Nigeria	AFR	LMIC	0.495	Mixed	2016	Health check	HPLC	170	9	NS	6-59m	Yes	Yes
V7	Lima DB, et al.[7]	2018	Brazil	AMR	UMIC	0.566	Both	2006	Community-based	HPLC	3417	598	NS	6-59m	Yes	Yes
V8	Chyne D, et al.[8]	2017	India	SEAR	LMIC	0.515	Rural	2014	Community-based	Precoded special chromatography filter paper (Whatman® #1)	197	116	NS	1-5y	No	Yes
V9	Longvah T, et al.[9]	2017	India	SEAR	LMIC	0.504	Rural	2013	Community-based	HPLC	227	74	NS	1-5y	No	Yes
V10	Alaofè H, et al.[10]	2017	Benin	AFR	LMIC	0.318	Rural	2014	Community-based	HPLC+ELISA	626	210	NS	6-59m	No	No
V11	Rahman S, et al.[11]	2017	Bangladesh	SEAR	LMIC	0.425	Both	2012	Community-based	HPLC	873	179	491	6-59m	Yes	No

ID	Author	Year Published	Country	WHO region	WB region	SDI	Setting	Study year	Survey base	Test	Tested sample	No. of VAD cases	No. of mVAD cases	Age range	Age-specific estimate	Sex-specific estimate
V12	Alouache A, et al.[12]	2017	Algeria	AFR	LMIC	0.593	Urban	2009	Community-based	HPLC	102	6	NS	41-72m	Yes	No
V13	Ayogu RN, et al.[13]	2016	Nigeria	AFR	LMIC	0.46	Urban	NS	School-based	TFA	50	22	NS	12-18y	Yes	No
V14	Wei X ,et al.[14]	2016	China	WPR	UMIC	0.638	Urban	NS	Community-based	HPLC	1928	53	274	7-11y	No	No
V15	Villalpando, et al.[15]	2015	Mexico	AMR	UMIC	0.617	Both	2012	Community-based	HPLC	5693	478	NS	1-11y	Yes	No
V16	Talsma EF, et al.[16]	2015	Kenya	AFR	LMIC	0.441	Rural	2010	School-based	HPLC	372	68	NS	6-12y	No	No
V17	Marasinghe E, et al.[17]	2015	Sri Lanka	SEAR	LMIC	0.636	Urban	NS	Community-based	HPLC	340	130	NS	2-5y	No	Yes
V18	Sandjaja, et al.[18]	2015	Indonesia	SEAR	LMIC	0.601	Rural	2011	Community-based	HPLC	504	77	NS	6m-12y	No	No
V19	Faber M, et al.[19]	2015	South Africa	AFR	UMIC	0.647	Both	2011	Community-based	HPLC	747	78	NS	1.5-6y	No	No
V20	Konstantyner T, et al.[20]	2014	Brazil	AMR	UMIC	0.566	Both	2006	Community-based	NS	1396	225	NS	0-2y	Yes	Yes
V21	Herrador Z, et al.[21]	2014	Ethiopia	AFR	LIC	0.233	Both	2009	Community-based	HPLC	663	194	NS	7-15y	No	No
V22	Ribeiro-Silva RC, et al.[22]	2014	Brazil	AMR	UMIC	0.583	Urban	2009	School-based	HPLC	546	49	103	7-14y	No	No
V23	Martínez-Torres J, et al.[23]	2014	Colombia	AMR	UMIC	0.574	Both	2010	Community-based	HPLC	4244	1151	NS	12-59m	Yes	Yes
V24	Dong C, et al.[24]	2014	China	WPR	UMIC	0.611	Rural	2009	Community-based	HPLC	268	32	50	24-60m	Yes	Yes
V25	Rohner F, et al.[25]	2014	Côte d'Ivoire	AFR	LMIC	0.333	Both	2007	Community-based	ELISA	782	188	NS	5-59m	No	No

ID	Author	Year Published	Country	WHO region	WB region	SDI	Setting	Study year	Survey base	Test	Tested sample	No. of VAD cases	No. of mVAD cases	Age range	Age-specific estimate	Sex-specific estimate
V26	Rojroongwasinkul N, et al.[26]	2013	Thailand	SEAR	UMIC	0.643	Both	2011	Community-based	HPLC	628	212	17	0.5-12.9y	Yes	No
V27	Poh BK, et al.[27]	2013	Malaysia	WPR	UMIC	0.698	Both	2011	Community-based	HPLC	1765	77	NS	6m-12y	Yes	Yes
V28	Queiroz D, et al.[28]	2013	Brazil	AMR	UMIC	0.572	Urban	2007	Community-based	HPLC	1211	265	652	6-59m	Yes	Yes
V29	Laillou A, et al.[29]	2012	Vietnam	WPR	LMIC	0.549	Both	2010	Community-based	HPLC	546	55	258	6-75m	Yes	No
V30	Hotz C, et al.[30]	2012	Zambia	AFR	LMIC	0.407	Rural	2009	Community-based	HPLC	574	273	NS	2-5y	No	No
V31	Laxmaiah A, et al.[31]	2012	India	SEAR	LMIC	0.421	Rural	2004	Community-based	HPLC	71591	1647	NS	1-4y	Yes	Yes
V32	Van Stuijvenberg ME, et al.[32]	2012	South Africa	AFR	UMIC	0.632	Mixed	2008	Health check	HPLC	243	14	NS	1-6y	No	No
V33	Engle-Stone R, et al.[33]	2011	Cameroon	AFR	LMIC	0.39	Both	NS	Community-based	HPLC	838	277	NS	12-59m	No	Yes
V34	Temple VJ, et al.[34]	2011	Papua New Guinea	WPR	LMIC	0.348	Urban	2008	Health check	HPLC	132	35	37	6-59m	Yes	Yes
V35	Arlappa N, et al.[35]	2011	India	SEAR	LMIC	0.414	Rural	2003	Community-based	HPLC	590	354	NS	3-5y	Yes	Yes
V36	Arlappa N, et al.[36]	2011	India	SEAR	LMIC	0.414	Rural	2003	Community-based	HPLC	407	358	NS	1y-5y	Yes	Yes
V37	Fares S, et al.[37]	2011	Tunisia	EMR	LMIC	0.599	Both	NS	School-based	HPLC	6677	154	1135	5-7y	Yes	No
V38	Durán P, et al.[38]	2011	Argentina	AMR	UMIC	0.649	Mixed	2005	Community-based	HPLC	32474	4644	195	6m-5y	No	No

ID	Author	Year Published	Country	WHO region	WB region	SDI	Setting	Study year	Survey base	Test	Tested sample	No. of VAD cases	No. of mVAD cases	Age range	Age-specific estimate	Sex-specific estimate
V39	Dillon D, et al.[39]	2010	Indonesia	SEAR	LMIC	0.537	Mixed	2001	School-based	HPLC	54	10	NS	6-12y	No	Yes
V40	Custodio VI, et al.[40]	2009	Brazil	AMR	UMIC	0.556	Mixed	2004	Health check	HPLC	103	6	21	5.5-11y	Yes	Yes
V41	Khatib IMD, et al.[41]	2009	Jordan	EMR	UMIC	0.63	Rural	2004	Health check	HPLC	262	66	138	6-66m	No	Yes
V42	Tatala SR, et al.[42]	2008	Tanzania	AFR	LMIC	0.284	Mixed	1996	School-based	HPLC	798	255	28	7-14y	Yes	No
V43	Jiang JX, et al.[43]	2008	China	WPR	UMIC	0.525	Both	2000	Community-based	NS	1236	97	373	0-71m	Yes	No
V44	Calis JCJ, et al.[44]	2008	Malawi	AFR	LIC	0.26	Both	2003	Community-based	HPLC	262	172	NS	6-60m	No	No
V45	Al-Mekhlafi MH, et al.[45]	2007	Malaysia	WPR	UMIC	0.659	Rural	2004	Community-based	HPLC	281	71	NS	2-15y	Yes	Yes
V46	Yang R, et al.[46]	2007	China	WPR	UMIC	0.581	Both	2006	Community-based	HPLC	357	11	26	0-59m	No	Yes
V47	Khan NC, et al.[47]	2007	Vietnam	WPR	LMIC	0.493	Mixed	NS	Community-based	HPLC	1657	199	NS	0-5y	No	No
V48	Jiang J, et al.[48]	2006	China	WPR	UMIC	0.525	Both	2000	Community-based	HPLC	7826	957	3140	1-6y	Yes	NO
V49	Hix J, et al.[49]	2006	Cambodia	WPR	LMIC	0.328	Mixed	2001	Community-based	HPLC	359	80	NS	6-59m	NO	NO
V50	Maziya-Dixon B.B, et al.[50]	2006	Nigeria	AFR	LMIC	0.356	Both	2001	Community-based	HPLC	3099	882	NS	0-5y	NO	NO
V51	Thurlow R.A, et al.[51]	2005	Thailand	SEAR	UMIC	0.594	Rural	2002	School-based	HPLC	481	19	NS	6-12.9y	NO	NO

ID	Author	Year Published	Country	WHO region	WB region	SDI	Setting	Study year	Survey base	Test	Tested sample	No. of VAD cases	No. of mVAD cases	Age range	Age-specific estimate	Sex-specific estimate
V52	Santos M.A, et al.[52]	2005	Brazil	AMR	UMIC	0.543	Rural	NS	School-based	Spectrophotometric method	241	70	NS	6-14y	NO	NO
V53	Nasri I, et al.[53]	2004	Morocco	EMR	LMIC	0.409	Mixed	NS	Community-based	HPLC	1453	587	NS	6-72m	NO	NO
V54	Coles C.L, et al.[54]	2004	Israel	EUR	HIC	0.742	Mixed	1996	Health check	HPLC	117	18	47	0-18m	NO	NO
V55	Gamble M.V, et al.[55]	2004	Marshall Islands	WPR	UMIC	0.423	Mixed	1994	Community-based	HPLC	367	201	NS	1-5y	NO	NO
V56	Yamamura C.M, et al.[56]	2004	Micronesia	WPR	LMIC	0.465	Rural	1993	Community-based	HPLC	354	189	NS	24-48m	No	No
V57	Villalpando S, et al.[57]	2003	Mexico	AMR	UMIC	0.558	Both	1999	Community-based	HPLC	1709	NS	NS	1-12y	No	No
V58	Adelekan D.A, et al.[58]	2003	Nigeria	AFR	LMIC	0.334	Mixed	1997	Health check	HPLC	192	138	NS	1-20d	No	No
V59	Oso O.O, et al.[59]	2003	Nigeria	AFR	LMIC	0.315	Rural	1993	Health check	HPLC	213	159	NS	6-72m	Yes	No
V60	Palafox N.A, et al.[60]	2003	Marshall Islands	WPR	UMIC	0.43	Mixed	1995	Community-based	HPLC	904	543	NS	1-5y	No	No
V61	Tan Z, et al.[61]	2002	China	WPR	UMIC	0.525	Both	2000	Community-based	Fluorescence method	8669	1018	NS	0-6y	Yes	No
V62	Schémann J.F, et al.[62]	2002	Mali	AFR	LIC	0.151	Both	1997	Community-based	HPLC	192	178	NS	6-72m	No	No
V63	Swami H.M, et al.[63]	2001	India	SEAR	LMIC	0.393	Mixed	2000	Community-based	HPLC	190	49	NS	1-5y	No	No
V64	Centers for Disease	2001	Micronesia	WPR	LMIC	0.502	Rural	2000	Community-based	HPLC	485	237	NS	24-59m	No	No

ID	Author	Year Published	Country	WHO region	WB region	SDI	Setting	Study year	Survey base	Test	Tested sample	No. of VAD cases	No. of mVAD cases	Age range	Age-specific estimate	Sex-specific estimate
	Control Prevention[64]															
V65	Kassaye T, et al.[65]	2001	Ethiopia	AFR	LIC	0.161	Mixed	1997	Health check	HPLC	824	490	NS	6-9y	Yes	Yes
V66	Centers for Disease Control Prevention[66]	1999	Nepal	SEAR	LMIC	0.259	Mixed	1999	Health check	HPLC	190	49	NS	10-19y	NO	NO
V67	Ferraz I.S, et al.[67]	2000	Brazil	AMR	UMIC	0.524	Urban	1997	Health check	HPLC	103	22	NS	6-24m	Yes	Yes
V68	Li X.C, et al.[68]	2020	China	WPR	UMIC	0.611	Mixed	2009	Community-based	HPLC	688	194	NS	New-born	No	No
V69	Zhang Z, et al.[69]	2018	China	WPR	UMIC	0.659	Urban	2016	School-based	HPLC	2085	31	647	3-6y	No	No
V70	Gubert M.B, et al.[70]	2016	Brazil	AMR	UMIC	0.566	Both	2006	Community-based	HPLC	3425	506	NS	0-59m	No	No
V71	Jin C, et al.[71]	2016	China	WPR	UMIC	0.657	Both	2015	Health check	HPLC	1538	80	724	1m-16y	Yes	No
V72	Pajuelo J, et al.[72]	2015	Peru	AMR	UMIC	0.595	Both	2009	Community-based	HPLC	1465	175	NS	0-59m	Yes	Yes
V73	De Moura F.F, et al.[73]	2015	Nigeria	AFR	LMIC	0.451	Both	2011	Community-based	HPLC	549	93	NS	6-59m	No	No
V74	Kurihayashi A.Y, et al.[74]	2015	Brazil	AMR	UMIC	0.61	Mixed	2013	Health check	HPLC	84	10	5	2-7y	No	No
V75	Augusto R.A, et al.[75]	2015	Brazil	AMR	UMIC	0.572	Mixed	2007	School-based	HPLC	702	104	NS	4-10y	No	No

ID	Author	Year Published	Country	WHO region	WB region	SDI	Setting	Study year	Survey base	Test	Tested sample	No. of VAD cases	No. of mVAD cases	Age range	Age-specific estimate	Sex-specific estimate
V76	Peng R, et al.[76]	2014	China	WPR	UMIC	0.638	Mixed	2012	School-based	HPLC	492	44	214	3.69-5.41y	No	No
V77	de Paula W.K, et al.[77]	2014	Brazil	AMR	UMIC	0.566	Mixed	2006	Health check	HPLC	563	90	NS	6-59m	Yes	Yes
V78	Rohner F, et al.[78]	2013	Philippines	WPR	UMIC	0.558	Urban	2008	Community-based	HPLC	1784	146	NS	6-23m	No	No
V79	Miglioli T.C, et al.[79]	2013	Brazil	AMR	UMIC	0.566	Both	2006	Community-based	HPLC	608	42	NS	6-59m	Yes	Yes
V80	Fan P, et al.[80]	2012	China	WPR	UMIC	0.611	Rural	2009	Community-based	HPLC	254	103	83	6-24m	Yes	No
V81	Garcia M.T, et al.[81]	2011	Brazil	AMR	UMIC	0.572	Urban	2007	Community-based	HPLC	164	24	NS	6-24m	No	No
V82	Midyat L, et al.[82]	2011	Turkey	EUR	UMIC	0.641	Both	2006	Community-based	HPLC	101	20	NS	24-59m	NO	NO
V83	Azevedo M.M, et al.[83]	2010	Brazil	AMR	UMIC	0.572	Both	2007	School-based	HPLC	333	26	99	24-60m	NO	NO
V84	Demissie T, et al.[84]	2010	Ethiopia	AFR	LIC	0.203	Both	2006	Community-based	HPLC	965	329	NS	6-71m	NO	NO
V85	Khatib I.M, et al.[85]	2009	Jordan	EMR	UMIC	0.639	Rural	NS	School-based	HPLC	560	165	338	5.5-10y	NO	Yes
V86	Durán P, et al.[86]	2009	Argentina	AMR	UMIC	0.649	Mixed	2005	Community-based	HPLC	30514	4363	NS	6m-72m	NO	NO
V87	Maslova E, et al.[87]	2009	Colombia	AMR	UMIC	0.546	Mixed	2006	School-based	HPLC	2810	385	1189	5-12y	Yes	Yes
V88	Leal J.Y, et al.[88]	2007	Venezuela	AMR	UMIC	0.566	Both	2007	School-based	HPLC	73	5	30	13-19y	NO	NO
V89	Graebner I.T, et al.[89]	2007	Brazil	AMR	UMIC	0.551	Rural	2003	School-based	HPLC	155	52	NS	5-18y	Yes	NO

ID	Author	Year Published	Country	WHO region	WB region	SDI	Setting	Study year	Survey base	Test	Tested sample	No. of VAD cases	No. of mVAD cases	Age range	Age-specific estimate	Sex-specific estimate
V90	Poveda E, et al.[90]	2007	Colombia	AMR	UMIC	0.552	Both	2007	School-based	HPLC	267	12	96	2-5y	No	Yes
V91	de Souza V.D.S.L, et al.[91]	2007	Brazil	AMR	UMIC	0.547	Urban	2002	Community-based	HPLC	422	NS	42	7-17y	Yes	Yes
V92	Zhang Y, et al.[92]	2007	China	WPR	UMIC	0.543	Both	2002	Community-based	HPLC	1052	72	477	0-5y	Yes	Yes
V93	Faruque A.S, et al.[93]	2006	Bangladesh	SEAR	LMIC	0.324	Rural	1998	Community-based	HPLC	1302	255	466	2-6y	No	Yes
V94	Paiva A.A, et al.[94]	2006	Brazil	AMR	UMIC	0.547	Both	NS	School-based	HPLC	631	97	183	36-83m	No	Yes
V95	Li J.H, et al.[95]	2006	China	WPR	UMIC	0.543	Both	2002	Community-based	HPLC	380	32	170	3-12y	NO	NO
V96	Ahmed F, et al.[96]	2006	Bangladesh	SEAR	LMIC	0.336	Urban	2000	School-based	HPLC	381	6	84	11-16y	NO	Yes
V97	Ferraz I.S, et al.[97]	2005	Brazil	AMR	UMIC	0.538	Mixed	2000	Health check	HPLC	179	135	NS	2-6y	NO	NO
V98	Monge-Rojas R, et al.[98]	2005	India	SEAR	LMIC	0.4	Mixed	NS	School-based	HPLC	84	1	8	10-16y	NO	Yes
V99	Sibeko L.N, et al.[99]	2004	South Africa	AFR	UMIC	0.593	Urban	2000	Health check	HPLC	113	11	NS	1-6m	NO	NO
V100	Martins M.C, et al.[100]	2004	Brazil	AMR	UMIC	0.529	Both	1998	Community-based	HPLC	607	195	NS	6-60m	Yes	Yes
V101	Castejon H.V, et al.[101]	2004	Venezuela	AMR	UMIC	0.548	Urban	2004	Community-based	HPLC	202	44	NS	24-84m	Yes	NO
V102	Mi J, et al.[102]	2003	China	WPR	UMIC	0.534	Both	2001	Community-based	Microfluorescent spectrophotometery	1257	106	483	0-6y	Yes	NO

ID	Author	Year Published	Country	WHO region	WB region	SDI	Setting	Study year	Survey base	Test	Tested sample	No. of VAD cases	No. of mVAD cases	Age range	Age-specific estimate	Sex-specific estimate
V103	Lin L, et al.[103]	2002	China	WPR	UMIC	0.525	Both	2000	Community-based	HPLC	8669	1018	3396	0-6y	Yes	Yes
V104	Amaya-Castellanos D, et al.[104]	2002	Venezuela	AMR	UMIC	0.554	Both	NS	School-based	HPLC	207	46	51	24-85m	Yes	NO
V105	Oelofse A, et al.[105]	2002	South Africa	AFR	UMIC	0.584	Urban	1998	Community-based	HPLC	42	1	NS	6-12m	NO	NO
V106	Jinabhai C.C, et al.[106]	2001	South Africa	AFR	UMIC	0.58	Rural	NS	School-based	HPLC	335	116	132	8-10y	Yes	NO
V107	Dijkhuizen M.A, et al.[107]	2001	Indonesia	SEAR	LMIC	0.508	Rural	1996	Health check	HPLC	155	84	NS	2.4-10.5y	NO	NO
V108	Albalak R, et al.[108]	2000	Honduras	AMR	LMIC	0.363	Mixed	1996	Community-based	HPLC	1243	553	560	12-60m	Yes	NO
V109	Gowele VF, et al.[109]	2021	Tanzania	AFR	LMIC	0.399	Rural	2016	Community-based	HPLC	666	166	NS	5-10y	NO	NO
V110	Reddy GB, et al.[110]	2022	India	SEAR	LMIC	0.547	Both	2017	Community-based	NS	20122	3397		5-19y	Yes	Yes
V111	De Castro IRR, et al.[111]	2021	Brazil	AMR	UMIC	0.616	Urban	2014	Community-based	HPLC	536	68	NS	6-59m	Yes	NO
V112	Reddy GB, et al.[112]	2021	India	SEAR	LMIC	0.547	Both	2017	Community-based	NS	9563	1510	NS	1-5y	Yes	Yes
V113	Tian T, et al.[113]	2022	China	WPR	UMIC	0.669	Mixed	2017	Community-based	NS	3025	27	441	7-17y	NO	Yes
V114	Janmohamed A, et al.[114]	2020	Mongolia	WPR	LMIC	0.597	Mixed	2017	Community-based	NS	938	109	420	6-23m	NO	NO

ID	Author	Year Published	Country	WHO region	WB region	SDI	Setting	Study year	Survey base	Test	Tested sample	No. of VAD cases	No. of mVAD cases	Age range	Age-specific estimate	Sex-specific estimate
V115	Shan X, et al.[115]	2021	China	WPR	UMIC	0.659	Mixed	2016	Community-based	HPLC	3808	126	769	mean: 11.32y	NO	Yes
V116	Ernawati F, et al.[116]	2021	Indonesia	SEAR	LMIC	0.601	Mixed	2011	Community-based	HPLC	1008	15	NS	mean: 31.7m	NO	NO

Note: WHO=the World Health Organization; AFR=African region; AMR=region of the Americas; SEAR=South-East Asia region; EUR=European region; EMR=Eastern Mediterranean region; WPR=Western Pacific region; WB region=the World Bank region; LMIC=Low- and Middle-Income Country; UMIC=Upper Middle-Income Country; SDI=Socio-demographic Index; NS= Not Stated; UFLC= Ultra High Performance Liquid Chromatography; HPLC=High-Performance Liquid Chromatography; ELISA=Enzyme-linked Immunosorbent Assay; TFA=Trifluoroacetic Acid; VAD= Vitamin A Deficiency; mVAD= Marginal Vitamin A Deficiency; d-day; y=year; m=month.

Table S6. Quality scores for assessing the risk of bias in the included articles (n=116)

ID	Author	Year Published	Quality score					
			Sample population	Sample size	Participation	Outcome assessment	Analytical methods	Total scores
V1	Disalvo L, et al.[1]	2019	2	1	2	2	2	9
V2	Onoja U S, et al.[2]	2019	2	1	2	2	2	9
V3	Hanson C, et al.[3]	2018	2	1	2	2	2	9
V4	Ayogu R, et al.[4]	2018	2	2	2	2	2	10
V5	Pouraram H et al.[5]	2018	2	2	2	2	2	10
V6	Abolurin OO, et al.[6]	2018	2	1	0	2	2	7
V7	Lima DB, et al.[7]	2018	2	2	0	2	2	8
V8	Chyne D, et al.[8]	2017	2	1	2	2	2	9
V9	Longvah T, et al.[9]	2017	2	2	2	2	2	10
V10	Alaofè H, et al.[10]	2017	2	1	2	2	2	9
V11	Rahman S, et al.[11]	2017	2	2	1	2	2	9
V12	Alouache A, et al.[12]	2017	1	1	2	2	1	7
V13	Ayogu RN, et al.[13]	2016	1	0	2	2	2	7
V14	Wei X, et al.[14]	2016	2	2	2	2	2	10
V15	Villalpando, et al.[15]	2015	1	0	2	1	2	6
V16	Talsma EF, et al.[16]	2015	2	1	2	2	2	9
V17	Marasinghe E, et al.[17]	2015	1	1	2	2	2	8
V18	Sandjaja, et al.[18]	2015	1	1	2	2	0	6
V19	Faber M, et al.[19]	2015	2	2	2	2	2	10
V20	Konstantyner T, et al.[20]	2014	2	1	2	1	2	8
V21	Herrador Z, et al.[21]	2014	2	1	2	2	2	9
V22	Ribeiro-Silva RC, et al.[22]	2014	2	2	2	2	2	10
V23	Martínez-Torres J, et al.[23]	2014	2	1	0	2	2	7
V24	Dong C, et al.[24]	2014	2	1	2	2	2	9
V25	Rohner F, et al.[25]	2014	2	1	2	2	1	8
V26	Rojroongwasinkul N, et al.[26]	2013	1	1	2	2	2	8
V27	Poh BK, et al.[27]	2013	2	2	2	2	1	9
V28	Queiroz D, et al.[28]	2013	1	1	2	2	2	8
V29	Laillou A, et al.[29]	2012	2	0	2	2	0	6

ID	Author	Year Published	Quality score					
			Sample population	Sample size	Participation	Outcome assessment	Analytical methods	Total scores
V30	Hotz C, et al.[30]	2012	2	1	0	2	2	7
V31	Laxmaiah A, et al.[31]	2012	2	1	2	1	2	5
V32	Van Stuijvenberg ME, et al.[32]	2012	2	1	2	2	2	9
V33	Engle-Stone R, et al.[33]	2011	1	0	2	2	2	7
V34	Temple VJ, et al.[34]	2011	2	1	2	2	2	9
V35	Arlappa N, et al.[35]	2011	2	1	2	2	1	8
V36	Arlappa N, et al.[36]	2011	1	0	2	2	2	7
V37	Fares S, et al.[37]	2011	2	1	2	2	2	9
V38	Durán P, et al.[38]	2011	2	1	1	2	1	7
V39	Dillon D, et al.[39]	2010	1	0	2	2	2	7
V40	Custodio VI, et al.[40]	2009	2	1	2	2	1	8
V41	Khatib IMD, et al.[41]	2009	2	1	2	2	2	9
V42	Tatala SR, et al.[42]	2008	2	1	2	2	2	9
V43	Jiang JX, et al.[43]	2008	2	2	2	2	1	9
V44	Calis JCJ, et al.[44]	2008	1	0	2	2	2	7
V45	Al-Mekhlafi MH, et al.[45]	2007	2	1	2	2	2	9
V46	Yang R, et al.[46]	2007	2	1	2	2	2	9
V47	Khan NC, et al.[47]	2007	2	1	2	2	2	9
V48	Jiang J, et al.[48]	2006	2	1	2	2	2	9
V49	Hix J, et al.[49]	2006	2	2	2	2	1	9
V50	Maziya-Dixon B.B, et al.[50]	2006	2	2	1	2	1	8
V51	Thurlow R.A, et al.[51]	2005	2	2	0	2	2	8
V52	Santos M.A, et al.[52]	2005	2	0	2	2	1	7
V53	Nasri I, et al.[53]	2004	1	1	0	1	1	4
V54	Coles C.L, et al.[54]	2004	1	2	2	2	2	9
V55	Gamble M.V, et al.[55]	2004	2	1	0	2	1	6
V56	Yamamura C.M, et al.[56]	2004	1	2	2	2	2	9
V57	Villalpando S, et al.[57]	2003	2	1	2	2	1	8
V58	Adelekan D.A, et al.[58]	2003	2	1	0	2	2	7
V59	Oso O.O, et al.[59]	2003	2	1	2	2	1	8

ID	Author	Year Published	Quality score					
			Sample population	Sample size	Participation	Outcome assessment	Analytical methods	Total scores
V60	Palafox N.A, et al.[60]	2003	1	1	2	2	1	7
V61	Tan Z, et al.[61]	2002	2	2	1	2	2	9
V62	Schémann J.F, et al.[62]	2002	2	2	0	2	1	7
V63	Swami H.M, et al.[63]	2001	2	2	0	2	1	7
V64	Centers for Disease Control Prevention[64]	2001	2	1	2	2	1	8
V65	Kassaye T, et al.[65]	2001	2	2	0	2	1	7
V66	Centers for Disease Control Prevention[66]	1999	2	1	0	1	1	5
V67	Ferraz I.S, et al.[67]	2000	2	1	0	2	1	6
V68	Li X.C, et al.[68]	2020	2	2	1	2	2	9
V69	Zhang Z, et al.[69]	2018	2	1	2	2	2	9
V70	Gubert M.B, et al.[70]	2016	2	1	1	2	1	7
V71	Jin C, et al.[71]	2016	2	1	2	2	1	8
V72	Pajuelo J, et al.[72]	2015	2	1	2	2	1	8
V73	De Moura F.F, et al.[73]	2015	2	1	2	2	2	9
V74	Kurihayashi A.Y, et al.[74]	2015	2	2	1	2	2	9
V75	Augusto R.A, et al.[75]	2015	2	1	2	2	2	9
V76	Peng R, et al.[76]	2014	2	1	2	2	2	9
V77	de Paula W.K, et al.[77]	2014	2	1	1	2	2	8
V78	Rohner F, et al.[78]	2013	2	1	2	2	2	9
V79	Miglioli T.C, et al.[79]	2013	2	1	2	1	1	7
V80	Fan P, et al.[80]	2012	2	1	2	2	1	8
V81	Garcia M.T, et al.[81]	2011	1	1	2	1	1	6
V82	Midyat L, et al.[82]	2011	2	0	0	2	1	5
V83	Azevedo M.M, et al.[83]	2010	2	2	2	2	1	9
V84	Demissie T, et al.[84]	2010	2	0	2	2	1	7
V85	Khatib I.M, et al.[85]	2009	2	1	1	2	1	7
V86	Durán P, et al.[86]	2009	1	1	1	1	1	5
V87	Maslova E, et al.[87]	2009	2	1	1	2	2	8

ID	Author	Year Published	Quality score					
			Sample population	Sample size	Participation	Outcome assessment	Analytical methods	Total scores
V88	Leal J.Y, et al.[88]	2007	1	1	1	2	0	5
V89	Graebner I.T, et al.[89]	2007	2	1	1	2	1	7
V90	Poveda E, et al.[90]	2007	2	0	1	2	2	7
V91	de Souza V.D.S.L, et al.[91]	2007	2	1	0	2	2	7
V92	Zhang Y, et al.[92]	2007	2	1	2	2	2	9
V93	Faruque A.S, et al.[93]	2006	2	1	1	2	1	7
V94	Paiva A.A, et al.[94]	2006	2	1	2	2	1	8
V95	Li J.H, et al.[95]	2006	1	0	2	2	2	7
V96	Ahmed F, et al.[96]	2006	2	1	2	2	2	9
V97	Ferraz I.S, et al.[97]	2005	1	1	0	2	1	5
V98	Monge-Rojas R, et al.[98]	2005	2	1	0	2	1	6
V99	Sibeko L.N, et al.[99]	2004	2	1	1	2	1	7
V100	Martins M.C, et al.[100]	2004	2	1	2	2	2	9
V101	Castejon H.V, et al.[101]	2004	2	2	0	2	1	7
V102	Mi J, et al.[102]	2003	2	1	1	2	1	7
V103	Lin L, et al.[103]	2002	2	1	2	2	1	8
V104	Amaya-Castellanos D, et al.[104]	2002	2	1	0	2	1	6
V105	Oelofse A, et al.[105]	2002	2	1	0	2	1	6
V106	Jinabhai C.C, et al.[106]	2001	2	1	0	2	1	6
V107	Dijkhuizen M.A, et al.[107]	2001	2	1	0	1	2	6
V108	Albalak R, et al.[108]	2000	2	1	0	2	1	6
V109	Gowele VF, et al.[109]	2021	2	1	0	2	2	7
V110	Reddy GB, et al.[110]	2022	2	1	0	2	2	7
V111	De Castro IRR, et al.[111]	2021	2	1	0	2	2	7
V112	Reddy GB, et al.[112]	2021	2	1	0	2	2	7
V113	Tian T, et al.[113]	2022	2	1	0	2	2	7
V114	Janmohamed A, et al.[114]	2020	2	1	0	2	2	7
V115	Shan X, et al.[115]	2021	2	1	0	2	2	7

ID	Author	Year Published	Quality score					
			Sample population	Sample size	Participation	Outcome assessment	Analytical methods	Total scores
V116	Ernawati F, at al.[116]	2021	2	1	0	2	2	7

Table S7. National prevalence of VAD and public health significance in 2019, by age group

Development region	WHO region	Country	0-5 years		6-12 years		13-18 years		0-18 years	
			Prevalence (%)	Significance	Prevalence (%)	Significance	Prevalence (%)	Significance	Prevalence (%)	Significance
Low SDI	Africa	Burundi	49.23 (37.05-62.01)	Severe	39.54 (28.60-51.98)	Severe	30.57 (21.26-42.03)	Severe	41.12 (30.10-53.45)	Severe
Low-middle SDI	Africa	Comoros	27.69 (22.62-33.45)	Severe	20.80 (16.70-25.61)	Moderate	15.19 (11.94-19.15)	Moderate	21.77 (17.55-26.69)	Moderate
Low-middle SDI	Eastern Mediterranean	Djibouti	23.59 (19.28-28.49)	Moderate	17.73 (14.26-21.82)	Moderate	12.88 (10.14-16.23)	Moderate	18.23 (14.70-22.37)	Moderate
Low SDI	Africa	Eritrea	32.78 (26.17-40.19)	Severe	24.98 (19.49-31.45)	Moderate	18.75 (14.27-24.25)	Moderate	25.92 (20.33-32.44)	Severe
Low SDI	Africa	Ethiopia	40.83 (31.68-50.86)	Severe	31.89 (24.01-41.10)	Severe	24.10 (17.59-32.17)	Moderate	32.86 (24.91-42.04)	Severe
Low-middle SDI	Africa	Kenya	22.84 (18.66-27.68)	Moderate	16.86 (13.58-20.77)	Moderate	12.20 (9.64-15.34)	Mild	17.50 (14.13-21.50)	Moderate
Low SDI	Africa	Madagascar	33.06 (26.40-40.53)	Severe	25.28 (19.72-31.80)	Moderate	18.78 (14.29-24.31)	Moderate	26.28 (20.62-32.87)	Severe
Low SDI	Africa	Malawi	36.36 (28.85-44.75)	Severe	27.94 (21.63-35.35)	Severe	20.90 (15.77-27.23)	Moderate	28.97 (22.56-36.43)	Severe
High-middle SDI	Africa	Mauritius	9.80 (6.49-14.55)	Mild	6.90 (4.52-10.41)	Mild	4.85 (3.13-7.43)	Mild	6.89 (4.51-10.37)	Mild
Low SDI	Africa	Mayotte	31.38 (25.16-38.37)	Severe	23.90 (18.75-29.96)	Moderate	17.80 (13.63-22.92)	Moderate	24.67 (19.44-30.77)	Moderate
Low SDI	Africa	Mozambique	43.44 (33.07-54.54)	Severe	34.45 (25.29-45.00)	Severe	26.42 (18.72-35.95)	Moderate	35.62 (26.41-46.11)	Severe
Low SDI	Africa	Réunion	26.45 (21.21-32.35)	Severe	20.18 (15.83-25.30)	Moderate	14.96 (11.45-19.27)	Moderate	20.39 (16.04-25.49)	Moderate
Low SDI	Africa	Rwanda	31.38 (25.43-38.10)	Severe	23.68 (18.83-29.40)	Moderate	17.46 (13.56-22.22)	Moderate	24.76 (19.77-30.58)	Moderate
High-middle SDI	Africa	Seychelles	8.59 (5.52-13.13)	Mild	6.09 (3.87-9.46)	Mild	4.26 (2.67-6.72)	Mild	6.43 (4.10-9.95)	Mild
Low SDI	Eastern Mediterranean	Somalia	68.47 (49.41-82.82)	Severe	59.92 (40.17-76.86)	Severe	50.72 (31.51-69.69)	Severe	60.76 (41.41-77.25)	Severe
Low SDI	Africa	South Sudan	38.58 (30.27-47.79)	Severe	29.91 (22.84-38.21)	Severe	22.48 (16.68-29.65)	Moderate	31.07 (23.89-39.4)	Severe
Low SDI	Africa	Uganda	33.41 (26.78-40.86)	Severe	25.53 (20.02-32)	Severe	18.93 (14.49-24.39)	Moderate	26.73 (21.08-33.30)	Severe

Development region	WHO region	Country	0-5 years		6-12 years		13-18 years		0-18 years	
			Prevalence (%)	Significance	Prevalence (%)	Significance	Prevalence (%)	Significance	Prevalence (%)	Significance
Low SDI	Africa	United Republic of Tanzania	30.50 (24.66-37.09)	Severe	23.12 (18.32-28.76)	Moderate	17.06 (13.22-21.77)	Moderate	24.29 (19.35-30.04)	Moderate
Low-middle SDI	Africa	Zambia	22.09 (18.06-26.75)	Moderate	16.34 (13.17-20.11)	Moderate	11.80 (9.33-14.83)	Mild	17.19 (13.89-21.08)	Moderate
Low-middle SDI	Africa	Zimbabwe	25.33 (20.75-30.57)	Severe	19.03 (15.36-23.37)	Moderate	13.82 (10.92-17.35)	Moderate	19.82 (16.04-24.25)	Moderate
Low-middle SDI	Africa	Angola	23.57 (19.31-28.44)	Moderate	17.63 (14.21-21.66)	Moderate	12.84 (10.14-16.13)	Moderate	18.72 (15.15-22.88)	Moderate
Low-middle SDI	Africa	Cameroon	22.52 (18.45-27.19)	Moderate	16.72 (13.5-20.53)	Moderate	12.12 (9.59-15.20)	Mild	17.62 (14.27-21.55)	Moderate
Low SDI	Africa	Central African Republic	46.72 (34.99-58.90)	Severe	37.55 (26.97-49.51)	Severe	29.23 (20.16-40.35)	Severe	38.30 (27.77-50.11)	Severe
Low SDI	Africa	Chad	53.88 (39.79-67.91)	Severe	44.05 (31.00-58.36)	Severe	34.74 (23.32-48.50)	Severe	45.42 (32.39-59.48)	Severe
Low-middle SDI	Africa	Congo	16.13 (12.74-20.21)	Moderate	11.76 (9.17-14.96)	Mild	8.39 (6.43-10.88)	Mild	12.46 (9.74-15.79)	Mild
Low SDI	Africa	Democratic Republic of the Congo	33.91 (26.88-41.75)	Severe	26.13 (20.21-33.08)	Severe	19.5 (14.69-25.43)	Moderate	27.51 (21.44-34.56)	Severe
Middle SDI	Africa	Equatorial Guinea	9.76 (6.67-14.06)	Mild	6.96 (4.70-10.18)	Mild	4.86 (3.24-7.23)	Mild	7.53 (5.10-10.96)	Mild
Middle SDI	Africa	Gabon	10.47 (7.46-14.47)	Mild	7.51 (5.29-10.54)	Mild	5.26 (3.65-7.52)	Mild	8.11 (5.73-11.33)	Mild
Low-middle SDI	Africa	Sao Tome and Principe	20.43 (16.71-24.71)	Moderate	15.10 (12.17-18.57)	Moderate	10.95 (8.66-13.75)	Mild	15.76 (12.74-19.33)	Moderate
Middle SDI	Africa	Botswana	12.17 (8.93-16.35)	Mild	8.75 (6.35-11.94)	Mild	6.17 (4.41-8.57)	Mild	9.18 (6.68-12.48)	Mild
Low-middle SDI	Africa	Eswatini	17.65 (13.82-22.29)	Moderate	12.75 (9.86-16.35)	Mild	9.13 (6.94-11.93)	Mild	13.24 (10.26-16.94)	Moderate
Low-middle SDI	Africa	Lesotho	22.95 (18.75-27.80)	Moderate	16.90 (13.61-20.81)	Moderate	12.17 (9.61-15.30)	Mild	17.57 (14.19-21.57)	Moderate
Middle SDI	Africa	Namibia	14.13 (10.66-18.49)	Moderate	10.21 (7.61-13.57)	Mild	7.21 (5.29-9.76)	Mild	10.81 (8.08-14.31)	Mild

Development region	WHO region	Country	0-5 years		6-12 years		13-18 years		0-18 years	
			Prevalence (%)	Significance	Prevalence (%)	Significance	Prevalence (%)	Significance	Prevalence (%)	Significance
Middle SDI	Africa	South Africa	10.19 (7.03-14.53)	Mild	7.27 (4.96-10.53)	Mild	5.10 (3.43-7.52)	Mild	7.63 (5.22-11.01)	Mild
Low SDI	Africa	Benin	36.91 (28.78-45.86)	Severe	28.74 (21.78-36.89)	Severe	21.67 (15.93-28.77)	Moderate	29.91 (22.84-38.08)	Severe
Low SDI	Africa	Burkina Faso	50.44 (37.51-63.61)	Severe	40.89 (29.08-54.06)	Severe	32.01 (21.79-44.45)	Severe	42.13 (30.32-55.11)	Severe
Low-middle SDI	Africa	Cabo Verde	19.12 (15.53-23.31)	Moderate	14.07 (11.27-17.43)	Moderate	10.11 (7.95-12.79)	Mild	14.50 (11.64-17.92)	Moderate
Low SDI	Africa	Côte d'Ivoire	30.61 (24.58-37.38)	Severe	23.32 (18.33-29.19)	Moderate	17.25 (13.23-22.17)	Moderate	24.36 (19.25-30.30)	Moderate
Low SDI	Africa	Gambia	30.53 (24.42-37.39)	Severe	23.37 (18.28-29.36)	Moderate	17.33 (13.21-22.38)	Moderate	24.59 (19.35-30.67)	Moderate
Low-middle SDI	Africa	Ghana	17.40 (13.86-21.62)	Moderate	12.71 (9.99-16.03)	Mild	9.06 (7.00-11.66)	Mild	13.37 (10.55-16.81)	Moderate
Low SDI	Africa	Guinea	41.31 (31.74-51.68)	Severe	32.49 (24.15-42.18)	Severe	24.74 (17.79-33.36)	Moderate	33.57 (25.17-43.22)	Severe
Low SDI	Africa	Guinea-Bissau	36.99 (28.89-45.93)	Severe	28.80 (21.87-36.92)	Severe	21.69 (15.99-28.75)	Moderate	29.97 (22.92-38.11)	Severe
Low SDI	Africa	Liberia	34.52 (27.19-42.68)	Severe	26.68 (20.46-33.97)	Severe	20.01 (14.93-26.29)	Moderate	27.63 (21.33-34.95)	Severe
Low SDI	Africa	Mali	47.88 (35.7-60.38)	Severe	38.71 (27.64-51.14)	Severe	30.25 (20.69-41.92)	Severe	40.06 (28.95-52.32)	Severe
Low-middle SDI	Africa	Mauritania	22.17 (18.15-26.79)	Moderate	16.45 (13.27-20.21)	Moderate	11.88 (9.40-14.91)	Mild	17.37 (14.06-21.25)	Moderate
Low SDI	Africa	Niger	64.66 (46.82-80.43)	Severe	54.73 (37.29-72.07)	Severe	44.64 (28.59-62.62)	Severe	56.31 (39.04-73.18)	Severe
Low-middle SDI	Africa	Nigeria	20.85 (17.00-25.32)	Moderate	15.39 (12.37-18.98)	Moderate	11.08 (8.74-13.95)	Mild	16.34 (13.19-20.08)	Moderate
Low SDI	Africa	Senegal	32.85 (26.13-40.37)	Severe	25.27 (19.64-31.90)	Moderate	18.82 (14.25-24.45)	Moderate	26.40 (20.64-33.09)	Severe
Low SDI	Africa	Sierra Leone	38.04 (29.58-47.34)	Severe	29.65 (22.38-38.15)	Severe	22.43 (16.43-29.86)	Moderate	30.61 (23.27-39.10)	Severe
Low SDI	Africa	Togo	30.44 (24.54-37.08)	Severe	23.12 (18.26-28.83)	Moderate	17.10 (13.19-21.88)	Moderate	24.10 (19.13-29.89)	Moderate
Middle SDI	Africa	Algeria	11.20 (8.03-15.41)	Mild	8.05 (5.7-11.24)	Mild	5.66 (3.95-8.04)	Mild	8.71 (6.19-12.11)	Mild

Development region	WHO region	Country	0-5 years		6-12 years		13-18 years		0-18 years	
			Prevalence (%)	Significance	Prevalence (%)	Significance	Prevalence (%)	Significance	Prevalence (%)	Significance
Middle SDI	Eastern Mediterranean	Egypt	11.92 (8.47-16.54)	Mild	8.62 (6.05-12.13)	Mild	6.01 (4.16-8.61)	Mild	9.19 (6.48-12.88)	Mild
High-middle SDI	Eastern Mediterranean	Libya	8.52 (5.61-12.72)	Mild	6.07 (3.95-9.21)	Mild	4.25 (2.73-6.55)	Mild	6.34 (4.14-9.58)	Mild
Low-middle SDI	Eastern Mediterranean	Morocco	17.63 (14.13-21.77)	Moderate	12.94 (10.25-16.23)	Moderate	9.24 (7.18-11.81)	Mild	13.42 (10.64-16.78)	Moderate
Low-middle SDI	Eastern Mediterranean	Sudan	21.86 (17.82-26.54)	Moderate	16.09 (12.94-19.86)	Moderate	11.60 (9.15-14.61)	Mild	16.88 (13.61-20.76)	Moderate
Middle SDI	Eastern Mediterranean	Tunisia	10.37 (7.22-14.66)	Mild	7.46 (5.13-10.71)	Mild	5.20 (3.53-7.6)	Mild	7.87 (5.43-11.26)	Mild
Middle SDI	Africa	Western Sahara	10.34 (7.33-14.38)	Mild	7.43 (5.20-10.49)	Mild	5.20 (3.59-7.47)	Mild	7.77 (5.46-10.94)	Mild
Middle SDI	Europe	Armenia	9.82 (6.67-14.23)	Mild	7.01 (4.71-10.33)	Mild	4.92 (3.26-7.36)	Mild	7.37 (4.96-10.81)	Mild
Middle SDI	Europe	Azerbaijan	10.27 (7.03-14.75)	Mild	7.40 (5.01-10.8)	Mild	5.14 (3.43-7.63)	Mild	7.82 (5.31-11.37)	Mild
High-middle SDI	Eastern Mediterranean	Bahrain	6.87 (4.22-10.96)	Mild	4.90 (2.98-7.93)	Mild	3.39 (2.04-5.59)	Mild	5.16 (3.15-8.32)	Mild
High-middle SDI	Europe	Georgia	9.37 (6.24-13.85)	Mild	6.71 (4.41-10.06)	Mild	4.68 (3.04-7.15)	Mild	7.06 (4.66-10.56)	Mild
Middle SDI	Eastern Mediterranean	Iraq	10.39 (7.24-14.67)	Mild	7.45 (5.13-10.68)	Mild	5.21 (3.54-7.61)	Mild	7.87 (5.44-11.24)	Mild
High-middle SDI	Europe	Israel	5.40 (3.04-9.40)	Mild	3.80 (2.12-6.72)	Mild	2.63 (1.45-4.72)	None	4.04 (2.26-7.10)	Mild
High-middle SDI	Eastern Mediterranean	Jordan	7.46 (4.74-11.53)	Mild	5.29 (3.33-8.31)	Mild	3.70 (2.30-5.91)	Mild	5.49 (3.46-8.59)	Mild
High-middle SDI	Eastern Mediterranean	Lebanon	8.34 (5.50-12.44)	Mild	5.93 (3.87-8.99)	Mild	4.14 (2.66-6.37)	Mild	6.14 (4.01-9.27)	Mild
High-middle SDI	Eastern Mediterranean	Oman	6.08 (3.54-10.23)	Mild	4.27 (2.47-7.30)	Mild	2.97 (1.69-5.15)	None	4.74 (2.74-8.04)	Mild
High-middle SDI	Eastern Mediterranean	Saudi Arabia	5.49 (3.08-9.59)	Mild	3.87 (2.15-6.87)	Mild	2.69 (1.48-4.83)	None	4.12 (2.30-7.27)	Mild
Low-middle SDI	Eastern Mediterranean	State of Palestine	14.45 (11.2-18.44)	Moderate	10.51 (8.04-13.61)	Mild	7.45 (5.61-9.83)	Mild	11.08 (8.50-14.30)	Mild
Middle SDI	Eastern Mediterranean	Syrian Arab Republic	13.64 (10.21-17.99)	Moderate	9.74 (7.20-13.06)	Mild	6.92 (5.03-9.44)	Mild	10.21 (7.56-13.63)	Mild

Development region	WHO region	Country	0-5 years		6-12 years		13-18 years		0-18 years	
			Prevalence (%)	Significance	Prevalence (%)	Significance	Prevalence (%)	Significance	Prevalence (%)	Significance
High-middle SDI	Europe	Turkey	7.28 (4.50-11.57)	Mild	5.15 (3.15-8.30)	Mild	3.58 (2.16-5.87)	Mild	5.32 (3.26-8.55)	Mild
Low SDI	Eastern Mediterranean	Yemen	31.34 (25.21-38.24)	Severe	23.89 (18.82-29.85)	Moderate	17.66 (13.58-22.66)	Moderate	24.76 (19.60-30.79)	Moderate
High-middle SDI	Europe	Kazakhstan	8.61 (5.54-13.14)	Mild	6.15 (3.92-9.54)	Mild	4.30 (2.71-6.78)	Mild	6.66 (4.26-10.27)	Mild
Low-middle SDI	Europe	Kyrgyzstan	15.69 (12.06-20.19)	Moderate	11.49 (8.73-15.00)	Mild	8.09 (6.04-10.77)	Mild	12.23 (9.32-15.91)	Mild
Low-middle SDI	Europe	Tajikistan	20.34 (16.4-24.98)	Moderate	14.98 (11.93-18.68)	Moderate	10.68 (8.34-13.58)	Mild	16.03 (12.81-19.90)	Moderate
Middle SDI	Europe	Turkmenistan	10.99 (7.67-15.50)	Mild	7.95 (5.49-11.38)	Mild	5.53 (3.76-8.06)	Mild	8.43 (5.84-12.02)	Mild
Middle SDI	Europe	Uzbekistan	13.12 (9.67-17.56)	Mild	9.44 (6.88-12.83)	Mild	6.65 (4.77-9.20)	Mild	10.00 (7.31-13.54)	Mild
Low SDI	Eastern Mediterranean	Afghanistan	40.32 (31.28-50.24)	Severe	31.47 (23.69-40.56)	Severe	23.86 (17.42-31.84)	Moderate	32.31 (24.48-41.36)	Severe
Low-middle SDI	South-East Asia	Bangladesh	24.37 (19.97-29.41)	Moderate	18.08 (14.59-22.20)	Moderate	13.13 (10.38-16.48)	Moderate	18.37 (14.84-22.52)	Moderate
Low-middle SDI	South-East Asia	Bhutan	26.72 (21.83-32.28)	Severe	19.92 (15.99-24.54)	Moderate	14.57 (11.45-18.38)	Moderate	20.20 (16.25-24.83)	Moderate
Low-middle SDI	South-East Asia	India	17.90 (14.15-22.39)	Moderate	12.95 (10.11-16.46)	Moderate	9.27 (7.11-12.00)	Mild	13.24 (10.35-16.79)	Moderate
Middle SDI	Eastern Mediterranean	Iran (Islamic Republic of)	10.32 (7.21-14.55)	Mild	7.35 (5.08-10.53)	Mild	5.16 (3.51-7.51)	Mild	7.83 (5.42-11.17)	Mild
Low-middle SDI	South-East Asia	Maldives	17.82 (14.14-22.23)	Moderate	13.06 (10.24-16.54)	Moderate	9.26 (7.13-11.96)	Mild	13.75 (10.80-17.35)	Moderate
Low SDI	South-East Asia	Nepal	31.75 (25.65-38.63)	Severe	23.99 (19.00-29.87)	Moderate	17.70 (13.69-22.61)	Moderate	24.11 (19.13-29.96)	Moderate
Low SDI	Eastern Mediterranean	Pakistan	27.70 (22.60-33.5)	Severe	20.80 (16.67-25.66)	Moderate	15.21 (11.93-19.21)	Moderate	21.64 (17.41-26.58)	Moderate
High-middle SDI	South-East Asia	Sri Lanka	11.13 (7.54-16.15)	Mild	7.89 (5.29-11.63)	Mild	5.55 (3.67-8.32)	Mild	8.17 (5.49-12.01)	Mild
Middle SDI	Western Pacific	China	10.01 (6.83-14.45)	Mild	7.13 (4.81-10.46)	Mild	5.00 (3.32-7.45)	Mild	7.39 (5.00-10.8)	Mild
Middle SDI	Western Pacific	China, Hong Kong SAR	8.94 (6.10-12.89)	Mild	6.31 (4.25-9.24)	Mild	4.40 (2.93-6.57)	Mild	6.71 (4.54-9.80)	Mild

Development region	WHO region	Country	0-5 years		6-12 years		13-18 years		0-18 years	
			Prevalence (%)	Significance	Prevalence (%)	Significance	Prevalence (%)	Significance	Prevalence (%)	Significance
Middle SDI	Western Pacific	China, Macao SAR	8.86 (6.04-12.79)	Mild	6.38 (4.31-9.35)	Mild	4.39 (2.92-6.55)	Mild	6.88 (4.65-10.03)	Mild
Low-middle SDI	South-East Asia	Dem. People's Republic of Korea	17.07 (13.59-21.22)	Moderate	12.40 (9.75-15.66)	Mild	8.84 (6.82-11.38)	Mild	12.67 (9.97-15.97)	Mild
Low-middle SDI	Western Pacific	Mongolia	13.75 (10.45-17.88)	Moderate	10.03 (7.53-13.23)	Mild	7.06 (5.22-9.50)	Mild	10.79 (8.13-14.18)	Mild
Low-middle SDI	Western Pacific	Cambodia	26.66 (21.83-32.17)	Severe	19.93 (16.06-24.50)	Moderate	14.51 (11.45-18.23)	Moderate	20.68 (16.72-25.33)	Moderate
Middle SDI	South-East Asia	Indonesia	11.35 (8.04-15.79)	Mild	8.14 (5.70-11.49)	Mild	5.71 (3.94-8.20)	Mild	8.41 (5.90-11.85)	Mild
Low-middle SDI	Western Pacific	Lao People's Democratic Republic	23.92 (19.59-28.88)	Moderate	17.71 (14.29-21.75)	Moderate	12.84 (10.16-16.12)	Moderate	18.30 (14.81-22.42)	Moderate
High-middle SDI	Western Pacific	Malaysia	7.65 (4.81-11.94)	Mild	5.39 (3.36-8.55)	Mild	3.75 (2.30-6.04)	Mild	5.58 (3.48-8.82)	Mild
Low-middle SDI	South-East Asia	Myanmar	21.58 (17.55-26.26)	Moderate	15.78 (12.66-19.52)	Moderate	11.39 (8.96-14.38)	Mild	16.04 (12.88-19.80)	Moderate
Middle SDI	Western Pacific	Philippines	13.61 (10.13-18.05)	Moderate	9.83 (7.23-13.24)	Mild	6.94 (5.03-9.52)	Mild	10.15 (7.48-13.63)	Mild
Middle SDI	South-East Asia	Thailand	10.27 (6.99-14.84)	Mild	7.28 (4.90-10.69)	Mild	5.11 (3.39-7.63)	Mild	7.40 (4.99-10.84)	Mild
Low-middle SDI	South-East Asia	Timor-Leste	22.26 (18.15-27.02)	Moderate	16.28 (13.09-20.08)	Moderate	11.75 (9.27-14.8)	Mild	16.92 (13.64-20.82)	Moderate
Middle SDI	Western Pacific	Viet Nam	14.44 (10.83-19.01)	Moderate	10.39 (7.70-13.89)	Mild	7.35 (5.36-10.00)	Mild	10.91 (8.10-14.53)	Mild
High-middle SDI	Americas	Antigua and Barbuda	8.68 (5.40-13.67)	Mild	6.13 (3.78-9.81)	Mild	4.26 (2.59-6.92)	Mild	6.38 (3.94-10.17)	Mild
Middle SDI	Americas	Aruba	13.45 (9.91-18)	Mild	9.50 (6.92-12.92)	Mild	6.75 (4.84-9.35)	Mild	9.66 (7.05-13.12)	Mild
High-middle SDI	Americas	Bahamas	5.78 (3.29-9.93)	Mild	4.00 (2.26-6.99)	Mild	2.80 (1.56-4.96)	None	4.09 (2.31-7.12)	Mild
High-middle SDI	Americas	Barbados	8.57 (5.35-13.47)	Mild	6.00 (3.70-9.59)	Mild	4.20 (2.56-6.82)	Mild	6.09 (3.76-9.71)	Mild
Middle SDI	Americas	Cuba	10.27 (7.19-14.44)	Mild	7.37 (5.10-10.52)	Mild	5.15 (3.52-7.49)	Mild	7.53 (5.22-10.73)	Mild

Development region	WHO region	Country	0-5 years		6-12 years		13-18 years		0-18 years	
			Prevalence (%)	Significance	Prevalence (%)	Significance	Prevalence (%)	Significance	Prevalence (%)	Significance
Middle SDI	Americas	Curaçao	11.55 (8.51-15.47)	Mild	8.33 (6.07-11.33)	Mild	5.89 (4.22-8.15)	Mild	8.44 (6.15-11.45)	Mild
Low-middle SDI	Americas	Dominican Republic	13.96 (10.78-17.89)	Moderate	10.09 (7.69-13.13)	Mild	7.16 (5.37-9.50)	Mild	10.44 (7.97-13.54)	Mild
Middle SDI	Americas	Grenada	11.59 (8.11-16.32)	Mild	8.28 (5.73-11.85)	Mild	5.81 (3.96-8.46)	Mild	8.70 (6.03-12.39)	Mild
Middle SDI	Americas	Guadeloupe	11.35 (8.37-15.20)	Mild	7.99 (5.81-10.87)	Mild	5.70 (4.09-7.89)	Mild	8.06 (5.88-10.95)	Mild
Low SDI	Americas	Haiti	27.70 (22.47-33.61)	Severe	20.92 (16.65-25.94)	Moderate	15.39 (11.98-19.56)	Moderate	21.48 (17.15-26.54)	Moderate
Middle SDI	Americas	Jamaica	10.26 (7.02-14.76)	Mild	7.31 (4.94-10.68)	Mild	5.10 (3.40-7.59)	Mild	7.53 (5.10-10.97)	Mild
Middle SDI	Americas	Martinique	11.71 (8.64-15.68)	Mild	8.24 (6.00-11.21)	Mild	5.88 (4.21-8.13)	Mild	8.30 (6.05-11.28)	Mild
Middle SDI	Americas	Saint Lucia	12.11 (8.46-17.08)	Mild	8.63 (5.95-12.36)	Mild	6.03 (4.10-8.78)	Mild	8.75 (6.05-12.51)	Mild
Middle SDI	Americas	Saint Vincent and the Grenadines	13.18 (9.76-17.56)	Mild	9.48 (6.94-12.83)	Mild	6.68 (4.82-9.21)	Mild	9.63 (7.06-13.01)	Mild
High-middle SDI	Americas	Trinidad and Tobago	7.49 (4.56-12.07)	Mild	5.29 (3.19-8.66)	Mild	3.69 (2.20-6.14)	Mild	5.48 (3.31-8.94)	Mild
High-middle SDI	Americas	United States Virgin Islands	5.41 (3.06-9.35)	Mild	3.81 (2.14-6.68)	Mild	2.65 (1.47-4.73)	None	3.90 (2.19-6.83)	Mild
Low-middle SDI	Americas	Belize	14.92 (11.37-19.33)	Moderate	10.75 (8.10-14.15)	Mild	7.61 (5.64-10.20)	Mild	11.07 (8.35-14.53)	Mild
Middle SDI	Americas	Costa Rica	9.68 (6.66-13.85)	Mild	6.89 (4.69-10.02)	Mild	4.83 (3.24-7.14)	Mild	7.10 (4.84-10.29)	Mild
Low-middle SDI	Americas	El Salvador	15.53 (12.21-19.54)	Moderate	11.25 (8.73-14.37)	Mild	8.01 (6.11-10.43)	Mild	11.55 (8.98-14.72)	Mild
Low-middle SDI	Americas	Guatemala	19.96 (16.20-24.35)	Moderate	14.64 (11.72-18.14)	Moderate	10.52 (8.26-13.30)	Mild	15.10 (12.11-18.66)	Moderate
Low-middle SDI	Americas	Honduras	21.95 (17.97-26.53)	Moderate	16.18 (13.06-19.89)	Moderate	11.73 (9.27-14.72)	Mild	16.54 (13.37-20.29)	Moderate
Middle SDI	Americas	Mexico	11.06 (7.96-15.16)	Mild	7.91 (5.63-11.01)	Mild	5.57 (3.90-7.89)	Mild	8.16 (5.81-11.32)	Mild

Development region	WHO region	Country	0-5 years		6-12 years		13-18 years		0-18 years	
			Prevalence (%)	Significance	Prevalence (%)	Significance	Prevalence (%)	Significance	Prevalence (%)	Significance
Low-middle SDI	Americas	Nicaragua	20.19 (16.45-24.54)	Moderate	14.88 (11.95-18.37)	Moderate	10.72 (8.45-13.51)	Mild	15.36 (12.36-18.92)	Moderate
Middle SDI	Americas	Panama	9.82 (6.70-14.17)	Mild	6.99 (4.71-10.24)	Mild	4.89 (3.25-7.30)	Mild	7.29 (4.92-10.64)	Mild
High-middle SDI	Americas	Argentina	8.27 (5.45-12.33)	Mild	5.87 (3.82-8.88)	Mild	4.10 (2.64-6.31)	Mild	6.10 (3.99-9.21)	Mild
Low-middle SDI	Americas	Bolivia (Plurinational State of)	16.13 (12.75-20.18)	Moderate	11.72 (9.15-14.89)	Mild	8.36 (6.42-10.83)	Mild	12.10 (9.46-15.33)	Mild
Middle SDI	Americas	Brazil	11.27 (8.21-15.26)	Mild	8.06 (5.80-11.08)	Mild	5.67 (4.02-7.94)	Mild	8.23 (5.93-11.30)	Mild
High-middle SDI	Americas	Chile	6.67 (4.04-10.78)	Mild	4.72 (2.83-7.74)	Mild	3.28 (1.94-5.47)	None	4.85 (2.92-7.95)	Mild
Middle SDI	Americas	Colombia	11.83 (8.70-15.88)	Mild	8.45 (6.14-11.52)	Mild	5.96 (4.26-8.28)	Mild	8.62 (6.27-11.73)	Mild
Middle SDI	Americas	Ecuador	12.12 (8.83-16.42)	Mild	8.68 (6.25-11.94)	Mild	6.11 (4.33-8.55)	Mild	9.02 (6.50-12.36)	Mild
High-middle SDI	Americas	French Guiana	8.03 (5.18-12.21)	Mild	5.65 (3.61-8.73)	Mild	3.94 (2.49-6.2)	Mild	5.98 (3.83-9.20)	Mild
Middle SDI	Americas	Guyana	14.78 (11.07-19.47)	Moderate	10.65 (7.88-14.25)	Mild	7.50 (5.46-10.22)	Mild	10.96 (8.12-14.63)	Mild
Middle SDI	Americas	Paraguay	12.30 (8.99-16.62)	Mild	8.80 (6.35-12.07)	Mild	6.21 (4.41-8.67)	Mild	9.13 (6.60-12.49)	Mild
Middle SDI	Americas	Peru	11.26 (8.12-15.40)	Mild	7.99 (5.69-11.11)	Mild	5.65 (3.97-8.00)	Mild	8.36 (5.97-11.58)	Mild
Middle SDI	Americas	Suriname	12.24 (8.96-16.49)	Mild	8.78 (6.35-12.01)	Mild	6.19 (4.41-8.62)	Mild	9.08 (6.58-12.39)	Mild
High-middle SDI	Americas	Uruguay	8.58 (5.75-12.58)	Mild	6.09 (4.04-9.07)	Mild	4.25 (2.78-6.45)	Mild	6.28 (4.17-9.33)	Mild
Low-middle SDI	Americas	Venezuela (Bolivarian Republic of)	12.79 (9.71-16.65)	Mild	9.29 (6.97-12.28)	Mild	6.59 (4.86-8.87)	Mild	9.50 (7.14-12.54)	Mild
Middle SDI	Western Pacific	Fiji	11.17 (7.87-15.62)	Mild	7.98 (5.56-11.33)	Mild	5.61 (3.85-8.11)	Mild	8.38 (5.85-11.86)	Mild
Low SDI	Western Pacific	New Caledonia	24.63 (20.1-29.78)	Severe	18.52 (14.86-22.83)	Moderate	13.54 (10.63-17.09)	Moderate	18.78 (15.09-23.10)	Moderate

Development region	WHO region	Country	0-5 years		6-12 years		13-18 years		0-18 years	
			Prevalence (%)	Significance	Prevalence (%)	Significance	Prevalence (%)	Significance	Prevalence (%)	Significance
Low SDI	Western Pacific	Papua New Guinea	35.59 (28.38-43.66)	Severe	27.23 (21.22-34.29)	Severe	20.26 (15.39-26.25)	Moderate	28.04 (21.96-35.14)	Severe
Low SDI	Western Pacific	Solomon Islands	33.09 (26.56-40.43)	Severe	25.30 (19.88-31.67)	Moderate	18.69 (14.33-24.04)	Moderate	26.47 (20.92-32.94)	Severe
Low-middle SDI	Western Pacific	Vanuatu	25.14 (20.60-30.34)	Severe	18.63 (15.04-22.88)	Moderate	13.58 (10.75-17.04)	Moderate	19.59 (15.87-23.97)	Moderate
Low-middle SDI	Western Pacific	Kiribati	19.68 (15.97-24.02)	Moderate	14.44 (11.56-17.90)	Moderate	10.41 (8.18-13.17)	Mild	15.28 (12.27-18.88)	Moderate
Low-middle SDI	Western Pacific	Micronesia (Fed. States of)	17.50 (13.67-22.16)	Moderate	12.64 (9.75-16.26)	Mild	9.00 (6.82-11.8)	Mild	13.09 (10.11-16.79)	Moderate
Low SDI	Western Pacific	French Polynesia	25.55 (20.86-30.88)	Severe	18.96 (15.21-23.39)	Moderate	13.98 (10.97-17.64)	Moderate	19.29 (15.50-23.73)	Moderate
Middle SDI	Western Pacific	Samoa	13.83 (10.06-18.74)	Moderate	9.87 (7.10-13.59)	Mild	6.98 (4.94-9.78)	Mild	10.54 (7.60-14.45)	Mild
Middle SDI	Western Pacific	Tonga	13.84 (10.13-18.64)	Moderate	9.93 (7.19-13.59)	Mild	7.02 (5.00-9.77)	Mild	10.30 (7.47-14.06)	Mild
High-middle SDI	Europe	Belarus	7.26 (4.51-11.48)	Mild	5.21 (3.20-8.35)	Mild	3.61 (2.19-5.88)	Mild	5.48 (3.38-8.76)	Mild
High-middle SDI	Europe	Bulgaria	6.80 (4.09-11.09)	Mild	4.77 (2.84-7.89)	Mild	3.35 (1.97-5.63)	None	4.94 (2.95-8.16)	Mild
High-middle SDI	Europe	Hungary	6.12 (3.51-10.43)	Mild	4.26 (2.42-7.38)	Mild	2.97 (1.67-5.22)	None	4.41 (2.51-7.60)	Mild
High-middle SDI	Europe	Poland	6.02 (3.39-10.46)	Mild	4.21 (2.35-7.42)	Mild	2.93 (1.62-5.25)	None	4.40 (2.46-7.73)	Mild
High-middle SDI	Europe	Republic of Moldova	10.10 (6.78-14.79)	Mild	7.2 (4.78-10.71)	Mild	5.04 (3.30-7.62)	Mild	7.45 (4.95-11.04)	Mild
High-middle SDI	Europe	Romania	7.41 (4.49-12.00)	Mild	5.18 (3.10-8.52)	Mild	3.63 (2.15-6.07)	Mild	5.35 (3.21-8.77)	Mild
High-middle SDI	Europe	Russian Federation	5.66 (3.17-9.88)	Mild	4.00 (2.22-7.08)	Mild	2.77 (1.52-4.98)	None	4.25 (2.37-7.51)	Mild
High-middle SDI	Europe	Ukraine	7.80 (4.91-12.15)	Mild	5.55 (3.46-8.79)	Mild	3.89 (2.39-6.25)	Mild	5.78 (3.61-9.12)	Mild
Middle SDI	Europe	Albania	10.21 (7.02-14.63)	Mild	7.28 (4.95-10.59)	Mild	5.07 (3.39-7.50)	Mild	7.39 (5.03-10.73)	Mild

Development region	WHO region	Country	0-5 years		6-12 years		13-18 years		0-18 years	
			Prevalence (%)	Significance	Prevalence (%)	Significance	Prevalence (%)	Significance	Prevalence (%)	Significance
High-middle SDI	Europe	Bosnia and Herzegovina	8.99 (5.83-13.61)	Mild	6.37 (4.09-9.80)	Mild	4.47 (2.83-6.98)	Mild	6.47 (4.15-9.93)	Mild
High-middle SDI	Europe	Croatia	6.26 (3.58-10.73)	Mild	4.40 (2.49-7.65)	Mild	3.06 (1.71-5.40)	None	4.53 (2.57-7.87)	Mild
High-middle SDI	Europe	Greece	5.85 (3.34-10.02)	Mild	4.09 (2.32-7.12)	Mild	2.86 (1.60-5.05)	None	4.15 (2.35-7.21)	Mild
High-middle SDI	Europe	Italy	5.81 (3.28-10.08)	Mild	4.08 (2.28-7.19)	Mild	2.84 (1.57-5.08)	None	4.16 (2.33-7.30)	Mild
High-middle SDI	Europe	Malta	5.41 (3.05-9.38)	Mild	3.80 (2.13-6.69)	Mild	2.63 (1.45-4.69)	None	3.97 (2.22-6.96)	Mild
High-middle SDI	Europe	Montenegro	6.19 (3.56-10.55)	Mild	4.32 (2.46-7.48)	Mild	3.01 (1.69-5.29)	None	4.47 (2.54-7.71)	Mild
High-middle SDI	Europe	North Macedonia	7.83 (4.87-12.35)	Mild	5.54 (3.41-8.87)	Mild	3.84 (2.34-6.26)	Mild	5.69 (3.51-9.10)	Mild
High-middle SDI	Europe	Portugal	7.67 (4.78-12.09)	Mild	5.40 (3.33-8.64)	Mild	3.78 (2.30-6.14)	Mild	5.46 (3.37-8.71)	Mild
High-middle SDI	Europe	Serbia	7.12 (4.26-11.67)	Mild	4.98 (2.95-8.29)	Mild	3.48 (2.04-5.88)	Mild	5.07 (3.01-8.42)	Mild
High-middle SDI	Europe	Spain	6.59 (3.94-10.79)	Mild	4.62 (2.74-7.69)	Mild	3.24 (1.90-5.48)	None	4.75 (2.82-7.88)	Mild

Note: SDI=Socio-demographic Index; WHO=the World Health Organization.

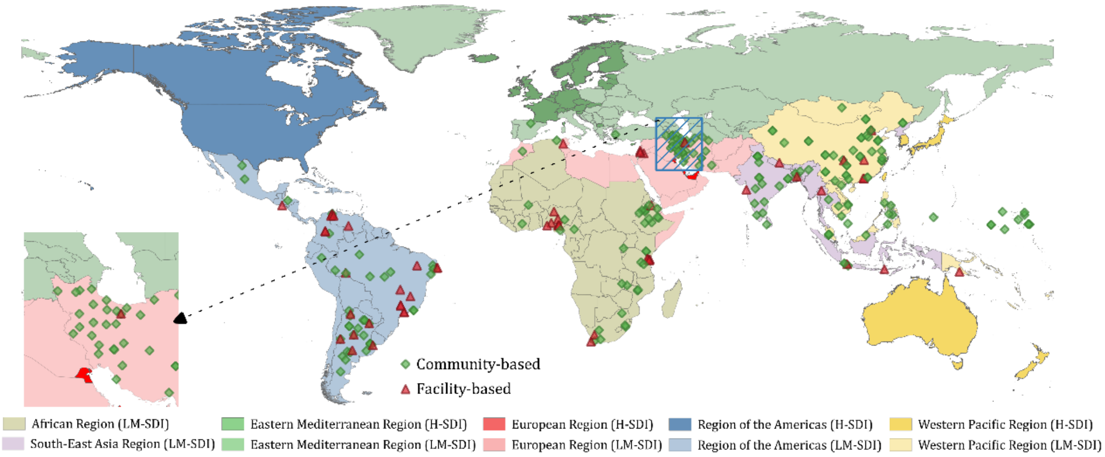

Figure S1. Contributing data sources across world regions

Note: H-SDI= high- socio-demographic index, including countries with SDI ⩾0.805; LM-SDI= low- and middle- socio-demographic index, including high-middle SDI, low-middle SDI, low SDI countries with an SDI <0.805.

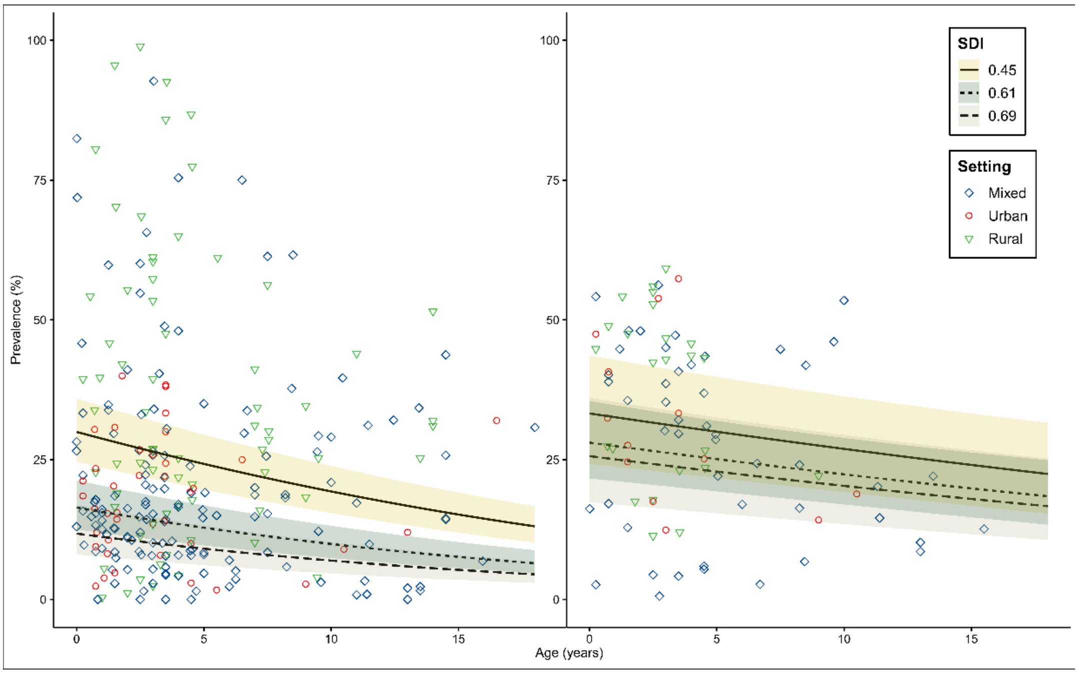

Figure S2. The relations of age and prevalence of VAD and mVAD, by SDI

Note: SDI=Socio-demographic Index; VAD= Vitamin A Deficiency; mVAD= Marginal Vitamin A Deficiency.

eMethods. The detailed description of stages in the national estimation of VAD or mVAD prevalence in LMICs

This section is a supplement to the Methods part in the main text.

Stage 1: Search strategy and selection criteria: identifying all epidemiological studies that reported prevalence of VAD/mVD in the general paediatric population using multiple data sources

Stage 2: Extracting data points of prevalence from the included studies

Stage 3: Epidemiological modelling of the prevalence of VAD and mVAD in children

Due to the hierarchical structure of extracted data, we applied a multilevel mixed-effects meta-regression approach to establish the regression models of VAD and mVAD prevalence. The effect of data points clustering from the same study was controlled by adding the study identification and country identification into the regression model as the random effect (u_i).[117]

Given that:

$$\text{prevalence} = \text{p} = \frac{(number\ of\ cases)}{(number\ of\ participants)}$$

Then, the prevalence was stabilized with the logit link,

$$\text{logit(p)} = \ln(\frac{p}{1-p}) = \ln(odds) = \alpha + \beta_1 * x_1 + \beta_2 * x_2 + \cdots \beta_n * x_n + u_i$$

Thus,

$$odds = \frac{p}{1-p} = e^{(\alpha+\beta_1*x_1+\beta_2*x_2+\cdots\beta_n*x_n+u_i)}$$

And,

$$prevalence = p = \frac{e^{(\alpha+\beta_1*x_1+\beta_2*x_2+\cdots\beta_n*x_n+u_i)}}{1+e^{(\alpha+\beta_1*x_1+\beta_2*x_2+\cdots\beta_n*x_n+u_i)}}$$

$x_1 - x_n$ were cluster-level variables, including age, sex (boys vs girls) or the proportion of girl (or the proportion of boy), setting (rural vs urban), investigation site (community-based or facility-based [school-based and health check-ups]), SDI (categorical as high-middle SDI vs low-middle and low SDI), WHO region, investigation period (categorical as before 2000, 2000-2009, 2010 and later), retinol test method. The associations of cluster-level variables were first examined using an age-adjusted meta-regression are shown in Table S4 in the **Online Supplementary Document**. The prevalence of VAD was found to be negatively associated with age and SDI, decreased in recent decades, and higher among boys (than among girls), in rural areas (than in urban areas), in low-middle

and low SDI region (than in high-middle SDI region), in AFR (than in WPR). While the prevalence of mVAD significantly decreased with increased age, and was higher in rural areas (than in urban areas). Bases on expertise, we included age and SDI as covariates in the regression models of VAD/mVAD prevalence. Therefore,

$$\text{logit(p)} = \alpha + \beta_1 * Age + \beta_2 * SDI + u_i$$

Then,

$$\text{prevalence of VAD/mVAD} = p = \frac{e^{(\alpha+\beta_1*Age+\beta_2*SDI+u_i)}}{1+e^{(\alpha+\beta_1*Age+\beta_2*SDI+u_i)}} = \frac{(number\ of\ VAD\ or\ mVAD\ cases)}{(number\ of\ participants)}$$

The graphical relation of age and prevalence of VAD and mVAD by SDI was demonstrated in Figure S2 in the **Online Supplementary Document**.

Stage 4: Estimation of the national prevalence of VAD and mVAD in children in 2019

Based on the above models, the age- and SDI-specific prevalence estimates of VAD and mVAD were respectively estimated. First, the total numbers of children affected by VAD and mVAD ("VAD envelope" and "mVAD envelope") in LMICs were respectively calculated by multiplying the estimated age- and SDI-specific prevalence rates by the corresponding paediatric populations in 2019, obtained from the United Nations Population Division.[118] As shown in Table S4 in the **Online Supplementary Document**, the prevalence of VAD and mVAD was higher in rural areas than in urban areas, to take into account this effect of urbanization, the odds ratios (OR) of being in urban areas (vs in rural areas) for VAD and mVAD as generated in stage 3 were obtained, and a risk-factor-based model was used to distribute the total numbers of children affected by VAD and mVAD in LMICs into the 165 LMIC nations[119,120] , by using the formula as follows:

$$N_{country} = (Pop_{country}) \times (Prev_{VAD/mVAD_{LMICs}}) \times [1 + (Prev_{urbanization_{country}} - Prev_{urbanization_{LMICs}}) \times (OR_{urbanization_{LMICs}} - 1)]$$

Where $N_{country}$ and $Pop_{country}$ are the number of VAD/mVAD cases and population size in children aged 18 years or younger in each country or territory. $Prev_{VAD/mVAD_{LMICs}}$ indicates the estimated prevalence of VAD or mVAD in LMICs where this country belongs. $Prev_{urbanization_{country}}$ and $Prev_{urbanization_{LMICs}}$ are the prevalence rates of urbanization in each country and in LMICs. $OR_{urbanization_{LMICs}}$ is the estimated OR of urbanization (urban areas vs rural areas) in LMICs. Finally,

the national prevalence of VAD and mVAD was generated by dividing the number of VAD and mVAD cases by the corresponding paediatric population in 2019.

References

1 Disalvo L, Biochemista AV, Biochemista NM, Biochemista AM, Fasano MV, González HFJAAP. Vitamin A deficiency and associated factors in preschoolers from the outskirts of La Plata, Buenos Aires. 2019;117:19-25.

2 Onoja U, Iloeje I, Onoja N, Uzor PJPJN. Nutritional status of adolescent schoolchildren in South East Nigeria. 2019;18:845-51.

3 Hanson C, Lyden E, Anderson-Berry A, Kocmich N, Rezac A, Delair S, et al. Status of retinoids and carotenoids and associations with clinical outcomes in maternal-infant pairs in Nigeria. 2018;10:1286.

4 Ayogu RN, Afiaenyi IC, Madukwe EU, Udenta EAJBph. Prevalence and predictors of under-nutrition among school children in a rural South-eastern Nigerian community: a cross sectional study. 2018;18:1-9.

5 Pouraram H, Djazayery A, Mohammad K, Parsaeian M, Abdollahi Z, Motlagh AD, et al. Second national integrated micronutrient survey in Iran: Study design and preliminary findings. 2018;21:137-44.

6 Abolurin OO, Adegbola AJ, Oyelami OA, Adegoke SA, Bolaji OOJNPMJ. Prevalence of vitamin A deficiency among under-five children in South-Western Nigeria. 2018;25:13.

7 Lima DB, Damiani LP, Fujimori EJRPdP. Vitamin A deficiency in Brazilian children and associated variables. 2018;36:176-85.

8 Chyne DAL, Meshram II, Rajendran A, Kodali V, Getti N, Roy P, et al. Nutritional status, food insecurity, and biodiversity among the K hasi in M eghalaya, N orth - East I ndia. 2017;13:e12557.

9 Longvah T, Khutsoh B, Meshram II, Krishna S, Kodali V, Roy P, et al. Mother and child nutrition among the Chakhesang tribe in the state of Nagaland, North - East India. 2017;13:e12558.

10 Alaofè H, Burney J, Naylor R, Taren DJPhn. Prevalence of anaemia, deficiencies of iron and vitamin A and their determinants in rural women and young children: a cross-sectional study in Kalalé district of northern Benin. 2017;20:1203-13.

11 Rahman S, Rahman AS, Alam N, Ahmed AS, Ireen S, Chowdhury IA, et al. Vitamin A deficiency and determinants of vitamin A status in Bangladeshi children and women: findings of a national survey. 2017;20:1114-25.

12 Alouache A, Hamma AS, Zenia S, Benlatreche CJCDNEDD. Status and intakes in vitamin A of a sample of young children Algerians. 2017;52:100-5.

13 Ayogu RN, Nnam NM, Ibemesi O, Okechukwu FJAHS. Prevalence and factors associated with anthropometric failure, vitamin A and iron deficiency among adolescents in a Nigerian urban community. 2016;16:389-98.

14 Wei X, Peng R, Cao J, Kang Y, Qu P, Liu Y, et al. Serum vitamin A status is associated with obesity and the metabolic syndrome among school-age children in Chongqing, China. 2016;25:563-70.

15 Villalpando S, de la Cruz V, Shamah-Levy T, Rebollar R, Contreras-Manzano AJSpdM. Nutritional status of iron, vitamin B12, folate, retinol and anemia in children 1 to 11 years old. Results of the Ensanut 2012. 2015;57:373-84.

16 Talsma EF, Verhoef H, Brouwer ID, Mburu-de Wagt AS, Hulshof PJ, Melse-Boonstra AJBm. Proxy markers of serum retinol concentration, used alone and in combination, to assess population vitamin A status in Kenyan children: a cross-sectional study. 2015;13:1-9.

17 Marasinghe E, Chackrewarthy S, Abeysena C, Rajindrajith SJAPjocn. Micronutrient status and its relationship with nutritional status in preschool children in urban Sri Lanka. 2015;24:144-51.

18 Sandjaja, Jus'at I, Jahari AB, Ifrad, Htet MK, Tilden RL, et al. Vitamin A-fortified cooking oil reduces vitamin A deficiency in infants, young children and women: results from a programme evaluation in Indonesia. Public health nutrition. 2015;18:2511-22.

19 Faber M, van Jaarsveld PJ, Kunneke E, Kruger HS, Schoeman SE, van Stuijvenberg MEJN. Vitamin A and anthropometric status of South African preschool children from four areas with known distinct eating patterns. 2015;31:64-71.
20 Konstantyner T, Warkentin S, de Aguiar Carrazedo Taddei JAJF, bulletin n. Prevalence and determinants of vitamin A deficiency among Brazilian children under 2 years of age from the 2006 National Demographic Health Survey. 2014;35:422-30.
21 Herrador Z, Sordo L, Gadisa E, Buño A, Gómez-Rioja R, Iturzaeta JM, et al. Micronutrient deficiencies and related factors in school-aged children in Ethiopia: a cross-sectional study in Libo Kemkem and Fogera districts, Amhara Regional State. 2014;9:e112858.
22 Ribeiro-Silva RdC, Nunes IL, Assis AMOJJdp. Prevalence and factors associated with vitamin A deficiency in children and adolescents. 2014;90:486-92.
23 Martínez-Torres J, Meneses-Echavéz JF, Ramírez-Vélez RJEyN. Prevalence of demographic factors associated with vitamin A deficiency in Colombian children aged 12–59 months. 2014;61:460-6.
24 Dong C, Ge P, Ren X, Zhao X, Wang J, Fan H, et al. The micronutrient status of children aged 24–60 months living in rural disaster areas one year after the Wenchuan earthquake. 2014;9:e88444.
25 Rohner F, Northrop-Clewes C, Tschannen AB, Bosso PE, Kouassi-Gohou V, Erhardt JG, et al. Prevalence and public health relevance of micronutrient deficiencies and undernutrition in pre-school children and women of reproductive age in Cote d'Ivoire, West Africa. 2014;17:2016-28.
26 Rojroongwasinkul N, Kijboonchoo K, Wimonpeerapattana W, Purttiponthanee S, Yamborisut U, Boonpraderm A, et al. SEANUTS: the nutritional status and dietary intakes of 0.5–12-year-old Thai children. 2013;110:S36-S44.
27 Poh BK, Ng BK, Haslinda MDS, Shanita SN, Wong JE, Budin SB, et al. Nutritional status and dietary intakes of children aged 6 months to 12 years: findings of the Nutrition Survey of Malaysian Children (SEANUTS Malaysia). 2013;110:S21-S35.
28 de Queiroz D, Paiva AdA, Pedraza DF, Cunha MALd, Esteves GH, de Luna JG, et al. Vitamin A deficiency and associated factors in children in urban areas. 2013;47:248-56.
29 Laillou A, Pham TV, Tran NT, Le HT, Wieringa F, Rohner F, et al. Micronutrient deficits are still public health issues among women and young children in Vietnam. 2012;7:e34906.
30 Hotz C, Chileshe J, Siamusantu W, Palaniappan U, Kafwembe EJPhn. Vitamin A intake and infection are associated with plasma retinol among pre-school children in rural Zambia. 2012;15:1688-96.
31 Laxmaiah A, Nair MK, Arlappa N, Raghu P, Balakrishna N, Rao KM, et al. Prevalence of ocular signs and subclinical vitamin A deficiency and its determinants among rural pre-school children in India. 2012;15:568-77.
32 Van Stuijvenberg ME, Schoeman SE, Lombard CJ, Dhansay MAJPHN. Serum retinol in 1–6-year-old children from a low socio-economic South African community with a high intake of liver: implications for blanket vitamin A supplementation. 2012;15:716-24.
33 Engle-Stone R, Haskell MJ, Ndjebayi AO, Nankap M, Erhardt JG, Gimou M-M, et al. Plasma retinol-binding protein predicts plasma retinol concentration in both infected and uninfected Cameroonian women and children. 2011;141:2233-41.
34 Temple VJ, Kaira C, Vince JD, Kevau IH, Willie NJPNGMJ. Vitamin A status of pre-school-age children aged 6 to 59 months in the National Capital District, Papua New Guinea. 2011;54:4-16.
35 Arlappa N, Laxmaiah A, Balakrishna N, Harikumar R, Kodavanti MR, Gal Reddy C, et al. Micronutrient deficiency disorders among the rural children of West Bengal, India. 2011;38:281-9.

36 Arlappa N, Balakrishna N, Laxmaiah A, Raghu P, Vikas Rao V, Madhavan Nair K, et al. Prevalence of vitamin A deficiency and its determinants among the rural pre-school children of Madhya Pradesh, India. 2011;38:131-6.
37 Fares S, Chahed MK, Feki M, Beji C, Traissac P, El Ati J, et al. Status of vitamins A and E in schoolchildren in the centre west of Tunisia: a population-based study. 2011;14:255-60.
38 Durán P, Mangialavori G, Biglieri A, Kogan L, Abeyá Gilardon EJRdlSBdP. Nutrition status in Argentinean children 6 to 72 months old: Results from the National Nutrition and Health Survey (ENNyS). 2011;50:30-43.
39 Dillon D, Htet MK, Chiwile FPJMJoI. The application of correction factors on serum retinol of Indonesia school children. 2010;19:258-63.
40 Custodio V, Daneluzzi JC, Custodio R, Del Ciampo LA, Ferraz I, Martinelli C, et al. Vitamin A deficiency among Brazilian school-aged children in a healthy child service. 2009;63:485-90.
41 Khatib IM, Elmadfa IJAoN, Metabolism. Poor nutritional health of Bedouin preschool children in Jordan: the irony of urbanization. 2009;54:301-9.
42 Tatala S, Kihamia C, Kyungu L, Svanbergrhaaa UJTjohr. Risk factors for anaemia in schoolchildren in Tanga Region, Tanzania. 2008;10.
43 Jiang J-X, Lin L-M, Lian G-L, Greiner TJWJoP. Vitamin A deficiency and child feeding in Beijing and Guizhou, China. 2008;4:20-5.
44 Calis JC, Phiri KS, Faragher EB, Brabin BJ, Bates I, Cuevas LE, et al. Severe anemia in Malawian children. The New England journal of medicine. 2008;358:888-99.
45 Al-Mekhlafi MH, Azlin M, Aini UN, Shaik A, Sa'iah A, Norhayati MJTotRSoTM, et al. Prevalence and predictors of low serum retinol and hypoalbuminaemia among children in rural Peninsular Malaysia. 2007;101:1233-40.
46 Yang R, Li R, Mao S, Sun L, Huang X, Ji C, et al. The survey of serum retinol of the children aged 0~ 4 years in Zhejiang Province, China. 2007;7:1-7.
47 Khan NC, Ninh NX, Nhien NV, Khoi HH, West CE, Hautvast JGJAPJoCN. Sub clinical vitamin A deficiency and anemia among Vietnamese children less than five years of age. 2007;16:152-7.
48 Jingxiong J, Toschke AM, von Kries R, Koletzko B, Liangming LJPhn. Vitamin A status among children in China. 2006;9:955-60.
49 Hix J, Rasca P, Morgan J, Denna S, Panagides D, Tam M, et al. Validation of a rapid enzyme immunoassay for the quantitation of retinol-binding protein to assess vitamin A status within populations. 2006;60:1299-303.
50 Maziya-Dixon BB, Akinyele IO, Sanusi RA, Oguntona TE, Nokoe SK, Harris EWJTJon. Vitamin A deficiency is prevalent in children less than 5 y of age in Nigeria. 2006;136:2255-61.
51 Thurlow RA, Winichagoon P, Green T, Wasantwisut E, Pongcharoen T, Bailey KB, et al. Only a small proportion of anemia in northeast Thai schoolchildren is associated with iron deficiency–. 2005;82:380-7.
52 Santos MA, Rezende EG, Lamounier JA, Galvão MAM, Bonomo É, Leite RCJRdN. Vitamin A deficiency in school children of the rural area in Minas Gerais, Brazil. 2005;18:331-9.
53 Nasri I, Bouhali E, Aguenaou H, Mokhtar NJAHS. Vitamin A deficiency among Moroccan women and children. 2004;4:3-8.
54 Coles C, Levy A, Gorodischer R, Dagan R, Deckelbaum R, Blaner W, et al. Subclinical vitamin A deficiency in Israeli-Bedouin toddlers. 2004;58:796-802.
55 Gamble M, Palafox N, Dancheck B, Ricks M, Briand K, Semba RJEjocn. Relationship of vitamin A deficiency, iron deficiency, and inflammation to anemia among preschool children in the Republic of the Marshall Islands. 2004;58:1396-401.
56 Yamamura CM, Sullivan KM, van der Haar F, Auerbach SB, Iohp KKJJoTP. Risk factors for vitamin A deficiency among preschool aged children in Pohnpei, Federated States of Micronesia. 2004;50:16-9.

57 Villalpando S, Montalvo-Velarde I, Zambrano N, García-Guerra A, Ramírez-Silva CI, Shamah-Levy T, et al. Vitamins A, and C and folate status in Mexican children under 12 years and women 12-49 years: a probabilistic national survey. 2003;45:508-19.
58 Adelekan DA, Northrop-Clewes CA, Owa JA, Oyedeji AO, Owoeye AA, Thurnham DIJBJoN. Use of biomarkers of sub-clinical infection, nutrition and neonatal maturity to interpret plasma retinol in Nigerian neonates. 2003;90:353-61.
59 Oso O, Abiodun P, Omotade O, Oyewole DJJotp. Vitamin A status and nutritional intake of carotenoids of preschool children in Ijaye Orile community in Nigeria. 2003;49:42-7.
60 Palafox NA, Gamble MV, Dancheck B, Ricks MO, Briand K, Semba RDJN. Vitamin A deficiency, iron deficiency, and anemia among preschool children in the Republic of the Marshall Islands. 2003;19:405-8.
61 Tan Z, Ma G, Lin L, Liu C, Liu Y, Jiang J, et al. Prevalence of subclinical vitamin A deficiency and its affecting factors in 8 669 children of China. 2002;36:161-3.
62 Schémann J-F, Banou AA, Guindo A, Joret V, Traore L, Malvy DJJotACoN. Prevalence of undernutrition and vitamin A deficiency in the Dogon Region, Mali. 2002;21:381-7.
63 Swami H, Thakur J, Bhatia S, Ahuja RJTIJoP. Rapid assessment and delivery of Vitamin A to slum children by using National Immunization Day in Chandigarh. 2001;68:719-23.
64 Control CfD, Prevention. Vitamin A deficiency among children--Federated States of Micronesia, 2000. 2001;50:509-12.
65 Kassaye T, Receveur O, Johns T, Becklake MRJBotWHO. Prevalence of vitamin A deficiency in children aged 6-9 years in Wukro, northern Ethiopia. 2001;79:415-22.
66 Control CfD, Prevention. Nutritional assessment of adolescent refugees--Nepal, 1999. 2000;49:864-7.
67 Ferraz I, Daneluzzi JC, Vannucchi HJNR. Vitamin A deficiency in children aged 6 to 24 months in S~ ao Paulo State, Brazil. 2000;20:757-68.
68 Li X, Zhou Y, Si K, Li H, Zhang L, Zhang Y, et al. Relationship of plasma vitamin A levels between neonates and pregnant women in third trimester. 2020;52:464-9.
69 Zhang Z-H, Ni M, Hu YJZDdekzzCJoCP. Current status of vitamin A deficiency in preschool children in Dongguan, China and the effect of vitamin A on serum ferritin and red blood cell parameters. 2018;20:195-9.
70 Gubert MB, Spaniol AM, Bortolini GA, Pérez-Escamilla RJPHN. Household food insecurity, nutritional status and morbidity in Brazilian children. 2016;19:2240-5.
71 Jin C, Wang X, Zhang L, Zhang Y, Li N, Wang J, et al. Analysis of fat-soluble vitamins A, D, E levels in the serum of healthy children. 2016;24:349-53.
72 Pajuelo J, Miranda M, Zamora R. Prevalence of vitamin a deficiency and anemia in children under five years of age in Peru. 2015.
73 De Moura FF, Moursi M, Lubowa A, Ha B, Boy E, Oguntona B, et al. Cassava intake and vitamin A status among women and preschool children in Akwa-Ibom, Nigeria. 2015;10:e0129436.
74 Kurihayashi AY, Augusto RA, Escaldelai FMD, Martini LAJCdSP. Vitamin A and D status among child participants in a food supplementation program. 2015;31:531-42.
75 Augusto RA, Cobayashi F, Cardoso MAJPHN. Associations between low consumption of fruits and vegetables and nutritional deficiencies in Brazilian schoolchildren. 2015;18:927-35.
76 Peng R, Wei X, Liang X, Chen J, Liu Y, Zhang T, et al. Effect of dietary vitamin A intake on plasma vitamin A concentration in preschool children of Banan district, Chongqing, China. 2014;46:366-72.
77 de Paula WKAS, Caminha MdC, Figueirôa JN, Batista Filho MJC, coletiva s. Anemia and vitamin A deficiency in children under five years old attended under the Family Health Program in the State of Pernambuco, Brazil. 2014;19:1209.

78 Rohner F, Bradley AW, Grant JA, Elizabeth AY, Lebanan MAO, Rayco-Solon P, et al. Infant and young child feeding practices in urban Philippines and their associations with stunting, anemia, and deficiencies of iron and vitamin A. 2013;34:S17-S34.
79 Miglioli TC, Fonseca VM, de Lira PIC, Batista Filho MJC, Coletiva S. Vitamin A deficiency in mothers and children in the state of Pernambuco. 2013;18:1427.
80 Fan P, Shen Y, Li Y, Ma A, Sun Z, Wang ZJWSyjJoHR. Serum retinol and carotenoid of rural infants and young children in Linyi of Shangdong Province and analysis on their related influencing dietary factors. 2012;41:424-8.
81 Garcia MT, Granado FS, Cardoso MAJCdSP. Complementary feeding and nutritional status of 6-24-month-old children in Acrelândia, Acre State, Western Brazilian Amazon. 2011;27:305-16.
82 Midyat L, Zafer Kurugöl N, Akşit S, Tanrıverdi M, Yağcı RVJAP. Dietary vitamin A intake and serum retinol concentrations of preschool children from different socio‐economical levels in İzmir, Turkey. 2011;100:e24-e7.
83 Sales de Azevedo MM, Coelho Cabral P, Diniz AdS, Fisberg M, Fisberg RM, Grande de Arruda IKJALdN. Vitamin A deficiency in preschool children of Recife, Northeast of Brazil. 2010;60:36-41.
84 Demissie T, Ali A, Mekonen Y, Haider J, Umeta MJF, bulletin n. Magnitude and distribution of vitamin A deficiency in Ethiopia. 2010;31:234-41.
85 Khatib IM, Elmadfa IJAoN, Metabolism. High prevalence rates of anemia, vitamin A deficiency and stunting imperil the health status of Bedouin schoolchildren in North Badia, Jordan. 2009;55:358-67.
86 Duran P, Mangialavori G, Biglieri A, Kogan LJAadp. Nutrition status in Argentinean children 6 to 72 months old: results from the National Nutrition and Health Survey (ENNyS). 2009;107:397-404.
87 Maslova E, Mora-Plazas M, Forero Y, López-Arana S, Baylin A, Villamor EJF, et al. Are vitamin A and iron deficiencies re-emerging in urban Latin America? A survey of schoolchildren in Bogota, Colombia. 2009;30:103-11.
88 Leal JY, Romero T, Ortega P, Amaya DJIC. Serum values of interleukin-10, gamma-interferon and vitamin A in female adolescents. 2007;48:317-26.
89 Graebner IT, Saito CH, de Souza EMJJdP. Biochemical assessment of vitamin A in schoolchildren from a rural community. 2007;83:247-52.
90 Poveda E, Cuartas A, Guarín S, Forero Y, Villarreal EJB. Iron and vitamin A micronutrient status, risk factors for their deficiencies and anthropometric assessment in preschool child from Funza municipality, Colombia. 2007;27:76-93.
91 da Silva LdSV, da Veiga GV, Ramalho RAJN. Association of serum concentrations of retinol and carotenoids with overweight in children and adolescents. 2007;23:392-7.
92 Zhang Y, Tao F, Yin H, Zhu X, Ji G, Kong S, et al. Breast-feeding, dietary intakes and their associations with subclinical vitamin A deficiency in children in Anhui Province, China. 2007;10:733-8.
93 Faruque AS, Khan A, Malek MA, Huq S, Wahed MA, Salam MA, et al. Childhood anemia and vitamin A deficiency in rural Bangladesh. 2006;37:771.
94 Paiva AdA, Rondó PHdC, Gonçalves-Carvalho CMR, Illison VK, Pereira JA, Vaz-de-Lima LRA, et al. Prevalence and factors associated with vitamin A deficiency in preschool children from Teresina, Piauí, Brazil. 2006;22:1979-87.
95 Li J, Piao J, Guo N, Yang LJWSyjJoHR. Study on the correlation of vitamin A and iron status in children. 2006;35:182-3.
96 Ahmed F, Rahman A, Noor AN, Akhtaruzzaman M, Hughes RJPhn. Anaemia and vitamin A status among adolescent schoolboys in Dhaka City, Bangladesh. 2006;9:345-50.
97 Ferraz IS, Daneluzzi JC, Vannucchi H, Jordão Jr AA, Ricco RG, Del Ciampo LA, et al. Prevalence of iron deficiency and its association with vitamin A deficiency in preschool children. 2005;81:169-74.

98 Monge-Rojas R, Barrantes M, Holst I, Nuñez-Rivas H, Alfaro T, Rodríguez S, et al. Biochemical indicators of nutritional status and dietary intake in Costa Rican Cabécar Indian adolescents. 2005;26:3-16.
99 Sibeko L, Dhansay M, Charlton KE, Johns T, Van Stuijvenberg M, Gray-Donald KJPhn. Full-term, peri-urban South African infants under 6 months of age are at risk for early-onset anaemia. 2004;7:813-20.
100 Martins MC, Santos LMP, Assis AMOJRdSP. Prevalence of hypovitaminosis A among preschool children from northeastern Brazil, 1998. 2004;38:537-42.
101 Castejon H, Ortega P, Amaya D, Gomez G, Leal J, Castejon OJJNN. Co-existence of anemia, vitamin A deficiency and growth retardation among children 24-84 months old in Maracaibo, Venezuela. 2004;7:113-9.
102 Mi J, Lin L, Ma G, Gu X, Liu M, Cheng H, et al. Prevalence of vitamin A deficiency in children under six years of age in Tibet, China. 2003;37:419-22.
103 Lin L, Liu Y, Ma G, Tan Z, Zhang X, Jiang J, et al. Survey on vitamin A deficiency in children under-6-years in China. 2002;36:315-9.
104 Amaya-Castellanos D, Viloria-Castejón H, Ortega P, Gómez G, Urrieta JR, Lobo P, et al. Vitamin A deficiency and the anthropometric nutritional status of urban and rural marginalized children in the state of Zulia, Venezuela. 2002;43:89-105.
105 Oelofse A, Van Raaij J, Benadé A, Dhansay M, Tolboom J, Hautvast JJPHN. Disadvantaged black and coloured infants in two urban communities in the Western Cape, South Africa differ in micronutrient status. 2002;5:289-94.
106 Jinabhai C, Taylor M, Coutsoudis A, Coovadia H, Tomkins A, Sullivan KJAoTP. A health and nutritional profile of rural school children in KwaZulu-Natal, South Africa. 2001;21:50-8.
107 Dijkhuizen MA, Wieringa FT, West CEJTAjocn. Concurrent micronutrient deficiencies in lactating mothers and their infants in Indonesia. 2001;73:786-91.
108 Albalak R, Ramakrishnan U, Stein AD, Van der Haar F, Haber MJ, Schroeder D, et al. Co-occurrence of nutrition problems in Honduran children. 2000;130:2271-3.
109 Gowele VF, Kinabo J, Jumbe T, Rybak C, Stuetz WJN. High prevalence of stunting and anaemia is associated with multiple micronutrient deficiencies in school children of small-scale farmers from Chamwino and Kilosa districts, Tanzania. 2021;13:1576.
110 Reddy GB, Shalini T, Ghosh S, Pullakhandam R, Kumar BN, Kulkarni B, et al. Prevalence of vitamin A deficiency and dietary inadequacy in Indian school-age children and adolescents. European journal of nutrition. 2022;61:197-209.
111 Castro IRR, Normando P, Alves-Santos NH, Bezerra FF, Citelli M, Pedrosa LFC, et al. Methodological aspects of the micronutrient assessment in the Brazilian National Survey on Child Nutrition (ENANI-2019): a population-based household survey. Cadernos de saude publica. 2021;3737:e00301120.
112 Reddy GB, Pullakhandam R, Ghosh S, Boiroju NK, Tattari S, Laxmaiah A, et al. Vitamin A deficiency among children younger than 5 y in India: an analysis of national data sets to reflect on the need for vitamin A supplementation. The American journal of clinical nutrition. 2021;113:939-47.
113 Tian T, Wang Y, Xie W, Zhang J, Ni Y, Peng X, et al. Multilevel Analysis of the Nutritional and Health Status among Children and Adolescents in Eastern China. Nutrients. 2022;14.
114 Janmohamed A, Luvsanjamba M, Norov B, Batsaikhan E, Jamiyan B, Blankenship JL. Complementary feeding practices and associated factors among Mongolian children 6-23 months of age. Maternal & child nutrition. 2020;16 Suppl 2:e12838.
115 Shan X, Liu C, Luo X, Zou Y, Huang L, Zhou W, et al. Iodine Nutritional Status and Related Factors among Chinese School-Age Children in Three Different Areas: A Cross-Sectional Study. Nutrients. 2021;13.
116 Ernawati F, Syauqy A, Arifin AY, Soekatri MYE, Sandjaja S. Micronutrient Deficiencies and Stunting Were Associated with Socioeconomic Status in Indonesian Children Aged 6-59 Months. Nutrients. 2021;13.

117 Hox JJ, Moerbeek M, Van de Schoot R. Multilevel analysis: Techniques and applications: Routledge; 2017.
118 United Nations, Department of Economic and Social Affairs, Population Division. World Population Prospects, the 2017 Revision. 2017. Available: https://esa.un.org/unpd/wpp/. Accessed April 13, 2022.
119 Song P, Xia W, Zhu Y, Wang M, Chang X, Jin S, et al. Prevalence of carotid atherosclerosis and carotid plaque in Chinese adults: A systematic review and meta-regression analysis. 2018;276:67-73.
120 Song P, Rudan D, Zhu Y, Fowkes FJ, Rahimi K, Fowkes FGR, et al. Global, regional, and national prevalence and risk factors for peripheral artery disease in 2015: an updated systematic review and analysis. 2019;7:e1020-e30.
